# GWATCH: a web platform for automated gene association discovery analysis

**DOI:** 10.1186/2047-217X-3-18

**Published:** 2014-11-05

**Authors:** Anton Svitin, Sergey Malov, Nikolay Cherkasov, Paul Geerts, Mikhail Rotkevich, Pavel Dobrynin, Andrey Shevchenko, Li Guan, Jennifer Troyer, Sher Hendrickson, Holli Hutcheson Dilks, Taras K Oleksyk, Sharyne Donfield, Edward Gomperts, Douglas A Jabs, Efe Sezgin, Mark Van Natta, P Richard Harrigan, Zabrina L Brumme, Stephen J O’Brien

**Affiliations:** 1 Theodosius Dobzhansky Center for Genome Bioinformatics, St. Petersburg State University, St. Petersburg 199004, Russia; 2 Department of Mathematics, St. Petersburg Electrotechnical University, St. Petersburg 197376, Russia; 3 Scientific Data Visualization Consultant, Turner, ACT 2612, Australia; 4 Genetics and Genomics Group, Advanced Technology Program, SAIC-Frederick, National Cancer Institute, Frederick, MD 21702, USA; 5 Department of Biology, Shepherd University, Shepherdstown, WV 25443, USA; 6 Vanderbilt Technologies for Advanced Genomics, Office of Research, Vanderbilt University Medical Center, Nashville, TN 37204, USA; 7 Biology Department, University of Puerto Rico, Mayaguez, PR 00680, USA; 8 Department of Biostatistics, Rho, Inc., Chapel Hill, NC 27517, USA; 9 Division of Hematology-Oncology, Children’s Hospital of Los Angeles, Los Angeles, CA 90027, USA; 10 Departments of Ophthalmology and Medicine, Icahn School of Medicine at Mount Sinai, New York, NY 10029, USA; 11 Department of Epidemiology, The Johns Hopkins University Bloomberg School of Public Health, Baltimore, MD 21205, USA; 12 British Columbia Centre for Excellence in HIV/AIDS, Vancouver, BC V6Z 1Y6, Canada; 13 Division of AIDS, Faculty of Medicine, University of British Columbia, Vancouver, BC V6T 1Z3, Canada; 14 Faculty of Health Sciences, Simon Fraser University, Burnaby, BC V5A 1S6, Canada; 15 Oceanographic Center, Nova Southeastern University, Ft. Lauderdale, FL 33004, USA

**Keywords:** AIDS, HIV, Complex diseases, Genome-wide association studies (GWAS), Whole genome sequencing (WGS)

## Abstract

**Background:**

As genome-wide sequence analyses for complex human disease determinants are expanding, it is increasingly necessary to develop strategies to promote discovery and validation of potential disease-gene associations.

**Findings:**

Here we present a dynamic web-based platform – GWATCH – that automates and facilitates four steps in genetic epidemiological discovery: 1) Rapid gene association search and discovery analysis of large genome-wide datasets; 2) Expanded visual display of gene associations for genome-wide variants (SNPs, indels, CNVs), including Manhattan plots, 2D and 3D snapshots of any gene region, and a dynamic genome browser illustrating gene association chromosomal regions; 3) Real-time validation/replication of candidate or putative genes suggested from other sources, limiting Bonferroni genome-wide association study (GWAS) penalties; 4) Open data release and sharing by eliminating privacy constraints (The National Human Genome Research Institute (NHGRI) Institutional Review Board (IRB), informed consent, The Health Insurance Portability and Accountability Act (HIPAA) of 1996 etc.) on unabridged results, which allows for open access comparative and meta-analysis.

**Conclusions:**

GWATCH is suitable for both GWAS and whole genome sequence association datasets. We illustrate the utility of GWATCH with three large genome-wide association studies for HIV-AIDS resistance genes screened in large multicenter cohorts; however, association datasets from any study can be uploaded and analyzed by GWATCH.

## Findings

### Introduction

Annotations of human genome variation have identified some 60 million single nucleotide polymorphisms (SNPs), which offer the promise of connecting nucleotide and structural variation to hereditary traits [1-3]. Genotyping arrays that resolve millions of common SNPs have enabled over 2,000 genome-wide associations studies (GWAS) to discover principal genetic determinants of complex multifactorial human diseases [4,5]. Today, whole-genome sequence association has extended the prospects for personalized genomic medicine, capturing rare variants, copy number variation (CNV), indels, epistatic and epigenetic interactions in hopes of achieving individualized genomic assessment, diagnostics, and therapy of complex maladies by interpreting one’s genomic heritage [6-9].

To date, GWAS studies have produced conflicting signals because many SNP associations are not replicated in subsequent studies. Further, GWAS frequently fail to implicate previously-validated gene regions described in candidate gene associations for the same disease, and in most cases offer less than 10% of the explanatory variance for the disease etiology [9-13]. In addition, discovered gene variants are frequently nested in noncoding desert regions of the genome that are difficult to interpret. At least part of these weaknesses derive from discounting SNP association “hits” that fail to achieve “genome-wide significance”, a widely accepted, albeit conservative, statistical threshold set to discard the plethora of false positive statistical associations (Type I errors) that derive from the large number of SNPs interrogated [2,13-16].

A challenge to genetic epidemiology involves disentangling the true functional associations that straddle the genome-wide significance threshold from the myriad of statistical artifacts that also occur. No one has developed a real solution to this conundrum, though some approaches have been offered [11,15-21]. Many researchers agree that more widely practiced open access data sharing of unabridged GWAS data would offer the opportunity for multiple plausible approaches to bear on this question [22,23]. However, for many cohorts, especially those developed before the advent of the genomics era, participants were not consented for open access of genome-wide data. Since patient anonymization is virtually impossible with genetic epidemiological data, the prospects of sharing patients’ genotype and clinical data may conflict with ethical concerns over protecting the individual privacy of study subjects [24-26]. GWATCH (Genome-Wide Association Tracks Chromosome Highway) addresses this issue through an organized open release of unabridged SNP-test association results from GWAS and whole genome sequencing (WGS) association studies and illustrates its utility using a SNP association analysis for HIV-AIDS in multiple cohorts [10,11,19,27-32].

## Results

GWATCH is a dynamic genome browser that automates and displays primary analysis results: p-values and Quantitative Association Statistic (QAS, a general term for statistics explaining direction and strength of associations: odds ratio, relative hazard and ez2-transformed correlation coefficient; see Section 2 of Additional file 1: Materials and Methods) from multiple association tests performed for one or more cohorts in a GWAS or WGS association study as a visual array ordered by SNP chromosomal position [33]. GWATCH offers a number of “features” that allow automated analysis and visualization of multiple test results, rapid discovery, replication and data release of unabridged association results (Table 1).

**Table 1:** Display feature components of GWATCH

**Features displayed**	**Illustration**
1. **Unabridged data table** of SNP chromosome coordinates, MAF*, p-value and QAS** for each SNP for each test	Additional file 2: Table S1
2. **Association tests list** and **Manhattan plots** for each test across all SNPs	Additional file 1: Figure S1
3. **SNAPSHOTS** of SNP-test results in a chromosome region:	
1. **2D heat plot snapshot** illustrating p-values in any selected chromosome region	Figure 1A and Additional file 1: Figure S2
2. **3D checkerboard plot snapshot** illustrating p-values and QAS** in any selected chromosome region	Figure 1B and Additiona file 1: Figure S3
3. **LD-polarized 3D checkerboard snapshot** illustrating p-values and QAS** in any selected chromosome region	Figure 1B and Additional file 1: Figure S4
4. **Dynamic HIGHWAY view by chromosome browser** illustrating p-values and QAS**	Figure 1C
5. **Top association hits:**	
1. **Top hits** based on **ranked -log p-value**	Additional file 10: Table S7
2. **Top hits** based on ranked QAS**	Additional file 10: Table S7
3. **Top hits** based on **ranked Density of -log p-value** within a SNP genomic region	Additional file 10: Table S7
6. **TRAX feature:**	
1. **TRAX PAGE** – two-page graphic summary illustrating p-values and QAS** for one selected SNP	Additional file 7: Figure S5
2. **TRAX REPORT** – eleven-page analysis summary with graphs, curves and tables for all association tests for one selected SNP	Additional file 8: Figure S6

Abbreviations:*MAF minor allele frequency, **QAS quantitative association statistic (OR, RH, ez2-transformed correlation coefficient).

**Figure 1:**
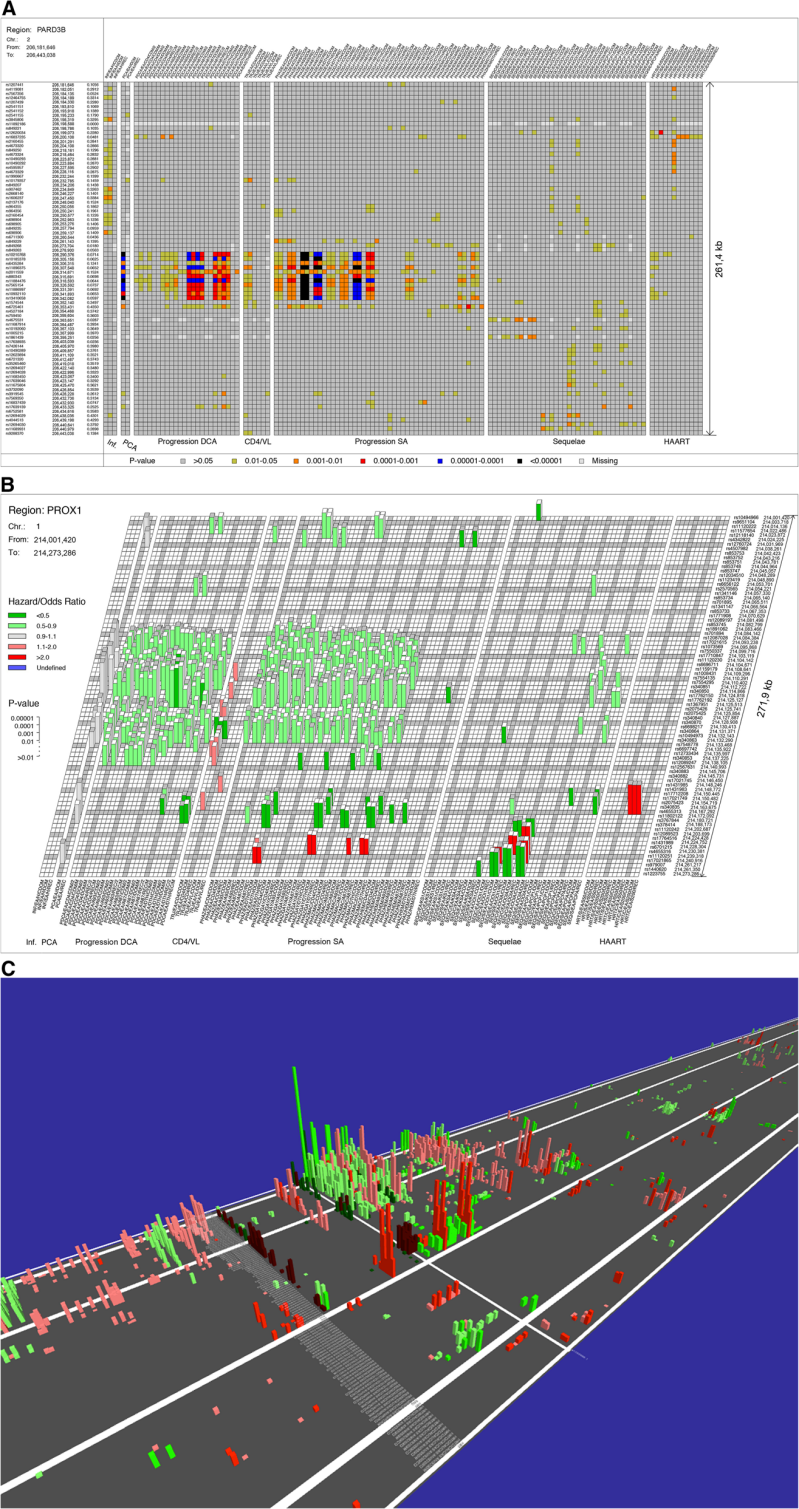
**GWATCH produces different kinds of snapshots and views for selected genomic region. (A)2D-SNAPSHOT** of *PARD3B* region of Chromosome 2 [28] tested for the 123 tests in Group A (Table 2). **(B) POLARIZED 3D-SNAPSHOT** of the *PROX1* region of Chromosome 1 [31] tested for the same group (Table 2). **(C)** Dynamic 3D **HIGHWAY** chromosome browser view of *CCR5* region of Chromosome 3 [32] tested for the same group (Table 2). See also in Additional file 1: Figures S2-S4.

A typical input of a GWAS analysis includes a large unabridged Data Table listing p-values and QASs across multiple SNP association tests performed for a list of ~10,000,000 ordered SNPs (Additional file 2: Table S1). GWATCH displays the Data Table, association tests and various perspectives for results: Manhattan plots for each single test (Additional File 1: Figure S1), 2D and 3D snapshots of test results for chromosome regions of “hits”, and a dynamic chromosome browser that illustrates significant p-values and QASs from the Data Table (Figure 1A, B and C). The imagery provides a dynamic traverse along a human chromosome producing a “bird’s eye” view of the strong SNP associations that rise above the chromosome highway surface. The idea is to visualize association results across a gene region (e.g., one that may include a highly significant SNP association) for all the tests performed (on the same or different cohorts) and for all the neighboring, potentially proxy SNPs (i.e., SNPs which track the neighboring causal, disease-affecting SNP due to the linkage disequilibrium [LD]) for the same tests.

Top hits are ranked based upon extreme p-values, QASs, or “density” of composite p-value peaks (representing proxy SNPs in linkage disequilibrium and multiple non-independent association tests). A multi-page “TRAX REPORT” produces curves, tables and appropriate statistics for a selected variant (SNP, indel or CNV tracked) on request. As genotyping and clinical data are organized, GWATCH automates the computation and visualization of results allowing instant replication of putative discoveries suggested by outside cohort studies or functional experiments. GWATCH also provides a simple procedure for web release of the association results to interested researchers.

We illustrate the utility, interpretation, and navigation of GWATCH using a GWAS carried out with study participants enrolled in eight prospective HIV-AIDS cohorts, searching for AIDS Restriction Genes [10,11,19,27-32]. We performed a GWAS meta-analysis on 5,922 patients with distinctive clinical outcomes genotyped using an Affymetrix 6.0 genotyping array (700,022 SNPs after quality control [QC] filters) and parsed into three population groups: Group A) A select group of 1,527 European American individuals; Group B) A larger group of 4,462 European American individuals that includes Group A; Group C) An independent group of 1,460 African American individuals (Table 2). Based upon available clinical information, we performed 123 association tests on Group A, 144 association tests on Group B, and 60 association tests on Group C (Table 3 and Additional file 3: Table S2, Additional file 4: Table S3, Additional file 5: Table S4, Additional file 6: Table S5). The tests include allele and genotype associations for four stages of AIDS: HIV acquisition/infection, AIDS progression (including categorical and survival analyses), AIDS-defining conditions and Highly Active AntiRetroviral Therapy (HAART) outcomes as described previously [27-32]; however, the unabridged dataset displayed in GWATCH-AIDS is far richer. For example, in references [28,31,32] each describes one association test (implicating the *PARD3B*, *PROX1*, and *CCR5-∆32* AIDS restriction genes respectively); [29,30] analyze small subsets of the SNPs tested within NEMP and HDF gene groups, respectively. GWATCH-AIDS presents complete results for 700,022 SNPs for 327 tests (Table 3) for 5,922 study participants listed in Table 2.

**Table 2:** Categories and numbers of patients genotyped in AIDS GWAS meta-analysis

**Abbreviations**	**Risk groups**	**Number of patients for each group**			**Total**
		**Group A**	**Group B**	**Group C**	**B + C**
		EA*-I	EA*-Total	AA**	
HREU	High Risk Exposed HIV Uninfected	254	300	148	448
EU (except HREU)	Exposed HIV Uninfected (all risks)	1	351	267	618
SC	Sero-Convertor	703	767	288	1 055
SP-LTS	Sero-Prevalent-Long-Term-Survivor (no AIDS for >10 years)	444	831	170	1 001
Sequelae	AIDS sequelae diagnosis	461	1 848	0	1 848
HAART	Anti-retroviral treatment	485	1 319	65	1 384
Total study participants		1 527	4 462	1 460	5 922

Abbreviations: *EA European Americans, **AA African Americans.

**Table 3:** **Statistical tests performed on 3 HIV-AIDS cohort Study Groups A-C (see Table **2**)**

**Clinical stage**	**Test type**	**Number of tests for each group**		
		**Group A**	**Group B**	**Group C**
I. HIV Infection	Ia. Infection - categorical	3	12	12
II. HIV Progression	IIa. Progression - categorical dichotomous	12	12	12
	IIb. Progression - categorical multipoint	12	12	12
	IIc. Progression - survival	48	48	24
III. AIDS-defining Conditions	IIIa. Sequelae - categorical first sequela	9	9	-
	IIIb. Sequelae - survival first sequela	9	-	-
	IIIc. Sequelae - categorical any sequelae	9	33	-
	IIId. Sequelae - survival any sequelae	9	6	-
IV. Treatment with ARV	IVa. HAART - categorical	6	-	-
	IVb. HAART - survival	6	12	-
Total		123	144	60

See Additional file 3: Table S2, Additional file 4: Table S3, Additional file 5: Table S4, Additional file 6: Table S5, for detailed description of statistical association tests performed in each group.

The first step of data analysis using GWATCH is to produce a large Data Table listing all SNP names, chromosome coordinates and minor allele frequency (MAF), with p-values and QASs for each test (Additional file 2: Table S1) plus a description of each test. Results in this Table are displayed as familiar Manhattan plots for each test as well as by **SNAPSHOT** views of chromosome regions. **2D-SNAPSHOT** is a heat plot of ordered SNP-test results (e.g., ~80 SNPs at 4 kb average distance for 123 tests in Group A (Table 2) equaling ~10,000 SNP-test combinations) indexed by the p-values from p > 0.05 (light grey) to richer colors for decreasing p-values, assuring that significant region clusters are more densely colorful (Figure 1A and Additional file 1: Figure S2). Similarly a **3D-SNAPSHOT** presents a checkerboard view of a chromosome region whereby the blocks rising above the surface reflect –log p-value and the color intensity reflects the QAS values with green indicating “resistant” associations (QAS < 1.0) and red showing “susceptible” ones (QAS > 1.0) (Figure 1B and Additional file 1: Figure S3 and S4). The moving browser **HIGHWAY**, a major feature of GWATCH, scrolls across the entire chromosomes in the 3D view of background statistical “noise” plus interesting regions of dense elevated blocks (Figure 1C).

Since susceptible/resistant colors are initially indexed by the minor (less common) allele at any locus, color discordance will arise in a region when minor allele at a given locus is tracked in LD by the common allele at an adjacent locus. The **POLARIZE** option corrects this computational artifact by inverting the QAS in locus pairs that show discrepant (common and minor allele tracking as proxies) LD polarity. When the entire association signal for a region, driving the non-independent SNPs and non-independent tests, derives from a single causal allele within the region, the blocks of associated SNPs in the viewed region should be the same color after polarization (Figure 1B and Additional file 1: Figure S4).

Automated searches for extreme locus “hits” revealing remarkable associations across the genome can be performed for each stage of disease (see above) screening for extreme p-values, QAS values and/or density of extreme p-values. For loci of particular interest, a detailed **TRAX REPORT** is generated to display each curve, table and statistic that had driven the association discovery (Additional file 7: Figure S5 and Additional file 8: Figure S6). TRAX REPORT is available for 641 SNPs in 241 genes listed in Additional file 9: Table S6. For the rest of the SNPs, the TRAX PAGE (shorter version of TRAX REPORT) is available.

To demonstrate GWATCH, three previously validated AIDS resistance gene regions, *CCR5-∆32, PROX1* and *PARD3B*, can be examined by simple entering rs-number, gene name or chromosome coordinates in the search option (see also 2D and 3D snapshots in Additional file 1: Figures S2-S4). GWATCH moves HIGHWAY to the selected region so one can visualize the signal with the 2D and 3D-SNAPSHOTS plus the TRAX REPORTS. Lastly, we also include a listing of discovered regions that showed AIDS association signals that, though they did not reach genome-wide significance, represented outlier values for several related tests and linked SNPs (Additional file 10: Table S7). These regions then would be considered as candidates for future evaluation and replication in independent cohort studies.

Finally, GWATCH is a generalizable web tool suitable for GWAS and/or WGS dataset for any complex disease. The “finished“or “processed” data (ones containing a final Data Table of p-values and QAS for completed association tests) can be uploaded directly by following instructions for dataset upload on the GWATCH website. “Primary” or “unfinished” data (ones with genotypes and clinical data for which tests need to be constructed and calculated) will be uploaded with our assistance in custom development of a disease-specific GWATCH-based analysis.

## Discussion

GWATCH is designed to enable investigators and users not connected to the original study to access the results of SNP association (from the whole genome sequence or SNP array genotyping) in order to view and share their study design and results openly. It can be used for visualization of regions with low p-values to inspect the pattern of variation across linked SNPs and also at different stages of disease (e.g., HIV infection, AIDS progression and treatment outcome).

As a primary discovery approach, screening across unabridged test results poses large statistical penalties for multiple tests eroding confidence in associations that fail to achieve genome-wide significance [2,13-18,21]. For this reason, one should use caution in inspection of putative regions of significance. Nonetheless, wholesale discarding of marginally significant “hits” will discount some true associations within the mix of statistical artifacts. GWATCH offers an opportunity to screen the genome for disease-associated regions, which may contain causal SNP variants included (or not) in the SNP array used for genotyping, as well as proxy SNPs tracking the causal variant. Further, in complex diseases for which there are many different cohorts being studied (e.g., in HIV-AIDS there are at least twenty different groups conducting AIDS GWAS on small, well-defined cohorts that may differ in genetic background and clinical data available for association testing) [34] GWATCH offers rapid replication opportunities with an independent dataset.

There are several websites that aim at cataloguing and displaying SNP associations. For example, GWAS Central [35] is a valuable resource for releasing and accessing GWAS data [36]. At the same time, we believe that GWATCH can be advantageous in some cases for the following reasons: 1) GWATCH utilizes (while not revealing directly) primary unabridged clinical/phenotypic data providing detailed analytical reports, like TRAX, not offered in GWAS Central; 2) GWATCH contains summary tools, such as top hits tables, and performs calculation of density that allows for identification, inspection and replication of putative association hits; 3) GWAS Central reports traditional Manhattan plots while GWATCH extends these to 2D and 3D static and dynamic region visuals that expands user comprehension and perception for better grasp of large data.

The GWATCH web browser provides a dynamic visual journey, similar to driving a video game along human chromosomes to view patterns of GWAS- or WGS-based variant association with any complex disease. It is meant to be appealing, intuitive, and accessible to non-experts and experts alike, including the various contributors to today’s exciting gene association studies. The format and open web access allows for importing new data from any disease-gene association study with multiple disease stages or genetic models of analysis. The wide breadth of test associations displayed is particularly suited to complex disease cohorts with detailed clinical parameters over distinct disorder stages. Further, although GWATCH is potentially useful for initial gene discovery, an important corollary lies in providing rapid replication of gene discoveries from independent cohort studies by simply keying in the putative gene region and inspecting the many test results of the posted dataset. Since replication screens are hypothesis-driven, they avoid the stringent multiple test correction penalties of a GWAS/WGS (p < 10^-8^). Finally, different cohort studies can be compared directly or combined to build meta-analyses.

Should many cohort investigators release their unabridged results, then association discoveries will be replicated (or not) in a rapid, open and productive manner, allowing for large meta-analyses as have been proposed for HIV-AIDS and other complex diseases [22,23,34]. Unlike other methods of data sharing, this results-based open data sharing/release approach avoids any violation of patient privacy, IRB (institutional review board) and HIPAA (Health Insurance Portability and Accountability Act of 1996) concerns, or informed consent constraints, since the primary clinical and genotype data remain confidential while the derivative results (p-values, QASs, plots) of multiple conceivable analytical approaches are openly released. In this approach, we hope to considerably expand discovery and replication opportunities in important biomedical research. To us, this ensures the maximum benefit of open access data sharing while protecting patients who prefer privacy (many do), but wish to see their volunteerism fulfilled.

## Materials and methods

### GWATCH implementation

GWATCH is a web-based application that integrates several technologies and programming languages. Server-side is represented by Apache web server, which employs PHP engine and Java-based toolkit Batik. R-project functions and modules are used for performing statistical tests, polarization and density calculation. MySQL database component of GWATCH allows access, retrieval and management of genotypes, clinical information and test results. On the frontend, GWATCH employs HTML5, Javascript, jQuery and WebGL for HIGHWAY browser interface, and Ajax and JSON technologies for data exchange between server and client.

### GWATCH tools

#### TRAX REPORTS

After screening for associations of clinical traits and genotypes one may be interested in a closer review of certain SNPs. The TRAX REPORT (Additional file 7: Figure S5 and Additional file 8: Figure S6) tool allows the production of reports on extended statistical analysis for any single SNP if the corresponding genotype and clinical information is available for all individuals. Important genotype information is given in the header on the TRAX front page: SNP identifier, SNP coordinate, chromosome, alleles and their frequencies. The header also lists information on populations involved in the analysis. In addition to the header, front page also contains a summary for all tests with p-values, as well as values of QAS represented in the bar plot form. The following pages of TRAX REPORT contain detailed information, such as contingency tables (that are produced in the form of corresponding bar plots for any categorical test, including progression categorical tests), and Kaplan–Meier survival curves that are reported for all three genotypes for all survival tests.

#### Polarization

The polarization tool enables the inversion of test results for minor and common SNP-alleles around some fixed SNP (called index SNP) for better approximation of true associations. A polarization table is produced using linkage disequilibrium coefficients (*D’*) between neighboring SNPs. Linkage disequilibrium coefficients are calculated for 80 SNPs upstream and 80 SNPs downstream of the index SNP. In the case of a sufficiently large positive value of linkage disequilibrium (*D’* > 0.9), the polarization mark is assigned to 1, whereas in the case of a sufficiently large negative linkage disequilibrium (*D’* < -0.9) the polarization mark is assigned to -1. If the linkage disequilibrium is sufficiently small, the polarization mark is assigned to 0. In the process of polarization, QAS values for test results of neighboring SNPs are inverted if the polarization mark is -1 implying the inversion of direction of disease association for such SNPs.

#### Density

Density top scoring that identifies regions of concentration of small p-values is calculated for each SNP in two steps:

1) in the window of specified size (*n* SNPs upstream and downstream or *n* Kbp upstream and downstream) average -log p-value is computed for each test (lane of the Highway)

2) these per-test (per-lane) averages are used for calculating density at this SNP either by averaging them or by finding the largest one (depending on the option chosen)

The second step can be performed for all the tests or for the group of tests by the disease stage (e.g., all tests for HIV infection, all tests for AIDS progression etc.).

### Statistical tests and data used for complex AIDS study

General types of statistical data and tests relevant to GWATCH are described in Additional file 1: Materials and Methods. Below we describe particular tests and data types used in the exemplary analysis of HIV/AIDS study data.

To illustrate GWATCH utility in the analysis of GWAS results we used data from multicenter longitudinal studies of several cohorts of patients exposed to the risk of HIV infection and/or already infected with HIV: ALIVE, DCG, HGDS, HOMER, LSOCA, MACS, MHCS and SFCC [11,34,37,38]. The total pool of patients was divided into three groups A, B and C based on ethnicity and timing of data development (see Table 2). A total of 5,922 patients were analyzed in all 3 groups.

All patient samples and genotypes were subjected to QC filtering depicted in Additional file 1: Table S8 as described previously [28,31]. Once final genotypes were obtained, population structure was assessed using the Principal Components Analysis module of *Eigensoft* software in European and African American populations [39] and structured SNP variants were excluded [28,39].

The statistical tests described below and listed in Table 3 and Additional file 3: Table S2, Additional file 4: Table S3, Additional file 5: Table S4 and Additional file 6: Table S5, were applied to the three patient study groups A, B and C (see Table 2). For each of the tests described below three genetic models were used (D, R and CD, see in Section 1 of Supplementary Materials and Methods under “Genotype classification” in Additional file 1) unless stated otherwise.

#### Infection tests (INF)

The aim of infection tests is to specify association of any selected genotype with HIV infection. The original clinical data is of categorical type based on the population of seronegatives (SN, individuals which stay HIV-negative throughout the whole study) at the baseline with the response variable indicating serostatus at the endpoint and having three levels: “high risk exposed uninfected” (HREU) seronegatives, “other seronegatives” (OSN) and “seroconverters” (SC, individuals which entered the study as HIV-negative, but became HIV-positive during the study). Three combinations of HIV status classifications were used to perform the categorical tests: “SC” vs. “HREU”, “SC” vs. “HREU” plus “OSN” and “SC” vs. “HREU” vs. “OSN”. In addition to the three genotype classifications described above (D, R and CD), allelic model (A) was also used for this test. One more group of individuals based on infection status, “seroprevalents” (SP, individuals which entered the study already being HIV-positive), was not informative for this type of test and therefore was not included in it.

#### Disease progression tests

The disease progression tests were used for screening significant associations between AIDS progression and genotype. The original data were of right-censored survival type under four different criteria of AIDS disease: CD4 < 200 (level of CD4+ cells falling below 200 cells/mm^3^), AIDS-1987 (patient meeting criteria of 1987 CDC definition of AIDS), AIDS-1993 (patient meeting criteria of 1993 CDC definition of AIDS) and Death from AIDS. Only SC and SP individuals were included in this analysis. SC individuals were included into analysis with HIV infection date (date of seroconvertion) as the baseline. SP individuals were included into categorical analysis with the date of the first visit as the baseline with some warnings.

*Disease progression categorical analysis* (PDCA) used the categorical tests for survival data (CTSD) approach described in Section 2 of Additional file 1: Materials and Methods. The CTSD were performed in dichotomous (PDCA2, two groups by the survival time or current status data) and multipoint (PDCAM, more than two groups by the survival time) forms. All individuals censored before the breakpoint were removed from the PDCA dichotomous analysis, as well as the SP individuals who failed before the breakpoint. All remaining individuals censored or failed after the breakpoint were classified into the group of long-term survivors (LTS, those who do not show AIDS symptoms before the breakpoint). The breakpoints used for classification in multipoint PDCA are stated in Additional file 3: Table S2, Additional file 4: Table S3, Additional file 5: Table S4.

*Proportional hazard (PHAZ*) analysis of disease progression used the proportional hazards survival tests (PHST) approach described in Section 2 of Additional file 1: Materials and Methods. These tests were performed for all four criteria of AIDS. Only SC individuals were included into PHAZ analysis.

#### Sequelae tests

Survival and categorical tests were performed for survival data on Kaposi’s sarcoma (KS), *Pneumocystis carnii* pneumonia (PCP), cytomegalovirus infection (CM), lymphoma (LY), mycobacterial infection (MYC) and other opportunistic infections (OOI). As in progression disease tests, survival sequelae tests included seroconverters only, while categorical sequelae tests included both seroconverters and seroprevalents.

*Sequelae tests for any infection order* classify patients based on whether specific sequela occurred at all, irrespectively of its order (i.e., whether it was the first sequela to occur for patient). The survival tests (SEQSA) under proportional hazards model as well as the progression categorical tests (SEQCA) were performed separately for each of the diseases described above.

*Sequelae tests for the first infection* classify patients based on whether specific sequela occurred first or not. The survival tests (SEQS1) under proportional hazards model as well as the progression categorical tests (SEQC1) were performed separately for each of the diseases described above.

#### Highly active antiretroviral therapy (HAART) tests

HAART tests were performed for the cohorts of patients who were subject to this type of treatment. Patients were classified based on either the level of suppression of HIV viral load or on the rebound of viral load following its suppression. Both survival (HRTS) and progression categorical (HRTC) tests were used for this analysis.

#### Hardy–Weinberg equilibrium (HWE) tests

The HWE tests are performed to control for the quality of data used for the screening of associations. Large deviations from HWE are not typical for the large populations and thus signal the genotyping error or some other type of data quality breach.

## Availability and requirements

**Project name:** GWATCH

**Project home page:** gen-watch.org

https://github.com/DobzhanskyCenter/GWATCH

**Operating system(s):** Platform independent (runs in the web browser)

**Programming language:** HTML5, Javascript, PHP, Java, R, MySQL

**Other requirements:** WebGL-supporting web browser (Firefox 4.0 and above; Chrome 12 and above; under OS X runs also in Safari 5.1 and above)

**License:** GPL v2.0

**Any restrictions to use by non-academics:** no

## Availability of supporting data

Archive of the version of GWATCH used in this paper is available from the *GigaScience* database [40], and for the most recent version please see our GitHub repository.

## Abbreviations

AIDS: Acquired immunodeficiency syndrome; CDC: Centers for Disease Control and Prevention; CNV: Copy-number variation; CTSD: categorical tests for survival data; GWAS: Genome-wide association study; GWATCH: Genome-Wide Association Tracks Chromosome Highway; HAART: Highly Active Antiretroviral Therapy; HIPAA: The Health Insurance Portability and Accountability Act of 1996; HIV: Human immunodeficiency virus; HREU: High risk exposed uninfected; HTML5: Hypertext markup language, revision 5; IRB: Institutional Review Board; HWE: Hardy-Weinberg equilibrium; LD: Linkage disequilibrium; LTS: Long-term survivor; MAF: Minor allele frequency; OSN: Other seronegatives; QAS: Quantitative Association Statistic; QC: Quality control; PDCA: Disease progression categorical analysis; PHAZ: Proportional hazard; PHP: Hypertext Preprocessor; SC: Seroconverter; SN: Seronegative; SNP: Single nucleotide polymorphism; SP: Seroprevalent; WGS: Whole genome sequencing.

## Competing interests

ASv, SM, NC, PG and SJO are authors of the provisional application for patent US 61/897,524 “Visualization, sharing and analysis of large data sets” filed on 10/30/2013.

## Authors’ contributions

ASv, SM, NC, PG, MR, PD, ASh, TKO and SJO developed GWATCH. LG, JT, SH, HHD, ES and SJO performed the original GWAS studies. SD, EG, DAJ, MVN, RH and ZLB contributed new epidemiological data from their AIDS cohorts. ASv, SM, NC and SJO wrote the manuscript. All authors read and approved the final manuscript.

## Supplementary Material

Additional file 1 **Supplementary Information.** Contains Materials and Methods, Figures S1–S4, legends for Figure S5 and S6, legends for Table S1–S7, Table S8 and References. Click here for file

Additional file 2: Table S1 Data Table of GWAS results: 100 rows of the Data Table containing SNPs, p-values and QASs for AIDS Restriction Genes dataset in Study Group A in the *PARD3B* region of chromosome 2. Full unabridged data tables for Groups A-C are available on the GWATCH web portal [33]. Click here for file

Additional file 3: Table S2 List of SNP association statistical tests and patient counts for Study Group A. Click here for file

Additional file 4: Table S3 List of SNP association statistical tests and patient counts for Study Group B. Click here for file

Additional file 5: Table S4 List of SNP association statistical tests and patient counts for Study Group C. Click here for file

Additional file 6: Table S5 Summary of SNP association tests performed for each Study Group. Click here for file

Additional file 7: Figure S5 TRAX PAGE, 2 page summary or all test results for a single SNP for a study group (e.g. p-values and QASs for HIV infection, AIDS progression using categorical and survival tests, AIDS sequelae, and HAART outcomes can be viewed and compared). TRAX PAGE can be generated *de novo* for any SNP of interest by placing mouse tip over a significant tower/block in the HIGHWAY and selecting the TRAX PAGE option from the data window that appears (SNPs for which TRAX REPORT is available do not have separate TRAX PAGE option in data window since TRAX REPORT includes TRAX PAGE content). Click here for file

Additional file 8: Figure S6 Detailed 11 page TRAX REPORT of derived statistics for all the tests accomplished including tables, bar graphs, survival curves and additional parameters for each test. TRAX REPORT can be generated *de novo* for the SNP of interest by placing mouse tip over a significant tower/block in HIGHWAY and selecting the TRAX REPORT option from the data window that appears. TRAX REPORTs are available for 641 SNPs in 241 human genes that were genotyped to replicate the GWAS associations for Study Groups A-C (Additional file 9: Table S6). Click here for file

Additional file 9: Table S6 List of 641 SNPs within 241 human genes that were assessed to replicate the GWAS associations for Study Groups A-C. For each of these SNPs a full TRAX REPORT (11 page report of figures and tables for each test) is available on the GWATCH web portal [33] as illustrated in Additional file 8: Figure S6. Click here for file

Additional file 10: Table S7 Genomic regions of remarkable statistical association (HITS) identified in ARG-GWAS by the screen for extreme p-values. Click here for file
