# Supplementary material for: GWATCH: a web platform for automated gene association discovery analysis
Source: Gigascience. 2014 Nov 5;3:18. doi: 10.1186/2047-217X-3-18 (PMC4220276; doi:10.1186/2047-217X-3-18)
Supplement: Additional file 1 — Supplementary Information. Contains Materials and Methods, Figures S1–S4, legends for Figure S5 and S6, legends for Table S1–S7, Table S8 and References. [file 2047-217X-3-18-S1.docx]

**GWATCH: a web platform for automated gene association discovery analysis**

Anton Svitin, Sergey Malov, Nikolay Cherkasov, Paul Geerts, Mikhail Rotkevich, Pavel Dobrynin, Andrey Shevchenko, Li Guan, Jennifer Troyer, Sher Hendrickson-Lambert, Holli Hutcheson Dilks, Taras K. Oleksyk, Sharyne Donfield, Edward Gomperts, Douglas A. Jabs, Mark Van Natta, P. Richard Harrigan, Zabrina L. Brumme, and Stephen J. O’Brien

**Supplementary Information:**

Supplementary Materials and Methods

Supplementary Figures 1-4

Legends for Supplementary Figures 5, 6

Legends for Supplementary Tables 1-7

Supplementary Table 8

Supplementary References

**Supplementary materials and** **methods**

Different tests for analysis of associations in the various types of data are included in GWATCH. A user having clinical and genotype data for one or several populations can select an appropriate group of tests for screening statistical associations of infection or disease progression with genotypes. The results of all selected statistical tests are visualized simultaneously in the HIGHWAY browser. Most of the statistical tests are executed using R-project. In addition, a special option enables display of detailed results of statistical analysis for any selected SNP via a TRAX REPORT.

**1. Statistical data**

Input data for the analysis consist of both clinical and genotype data. The genotype data contains information on all SNPs to be analyzed, and the corresponding genotype consists of two dummy (binary) variables specifying corresponding forms of two alleles or of one categorical variable with three levels specifying whole genotype information for the individual. In the former case, 0 corresponds to common SNP allele and 1 corresponds to minor SNP allele. In the latter case, common SNP homozygote is coded by -1, minor SNP homozygote is coded by 1, and heterozygote is coded by 0. The required SNP information is the SNP identifier (SNP ID) and the corresponding coordinate. The input genotype data are expected to be sorted by SNP coordinate sequentially. For further analysis all individuals will be subjected to different types of genotype classification.

*Genotype classification* is used as an explanatory factor for all statistical tests. Four types of genotype classification are used: dominant (D) classification separates common homozygote from all other genotypes in two different groups; recessive (R) classification separates minor homozygote from all other genotypes; and co-dominant (CD) classification separates all individuals into three groups by their genotype; under allelic classification (A), two SNP alleles corresponding to any single individual are considered as different observations with the same clinical data.

Two types of clinical data are acceptable for analysis: categorical and right-censored survival.

*Categorical data* consist of the ID variable and numeric categorical variable having two or more levels specifying disease status. In the case of two levels, it is recommended to use code 1 for affected individuals (i.e., individuals which acquire infection, demonstrate symptoms of disease etc.) and code 0 for other individuals. In the case of more than two levels and ordinal categories, it is recommended to use 0 for unaffected individuals (e.g., uninfected individuals or individuals with no disease symptoms etc.) and to choose positive numbers corresponding to other levels in the same order as the original categories.

*Right-censored survival data* contain information on the exact time from baseline date (preferentially in days) to an event and type of the event that is given by the binary variable: 1 corresponding to failure (event occurred) and 0 corresponding to censoring (no event), for any individual. For the competing risks model it is possible to use several positive levels for different types of failures.

**2. Statistical tools for associations**

GWATCH allows analysis of disease associations with genotype for all available SNPs. Tests corresponding to different genotype classifications can be produced for any clinical data by the selected testing method. Stratified analysis is available if the input clinical data contain a classification variable. In this case, any selected group of individuals is analyzed separately and the results of these tests are displayed on different lanes of the Highway.

*Categorical tests (CT)* (see [1]) are used for categorical statistical analysis of data organized as an m×k contingency table. The categorical data are required to perform categorical tests. Fisher’s exact test (**R**-function “fisher.test()”) for **2**×**2** contingency tables and chi-square test (**R**-function “chisq.test()”) are applied to produce p-value. The odds ratio for **2**×**2** contingency tables or the transformation of Pearson’s correlation coefficient (designated as ez2-transformation for the square of the exponentiated Fisher’s z-transformation) for the tables of other sizes define direction of the association, and therefore, color of the corresponding bar on the Highway.

*Proportional hazards survival tests (PHST)* are used for the analysis of right-censored survival data that are required for this type of tests. The Cox proportional hazards model [2] is used to produce p-value (**R**-function coxph(), package *survival*). The direction of the associations is defined by the obtained relative hazard, which is calculated as hazard ratio (for binary genotype classifications A, D and R) or exponentiated slope of Cox’s regression line (under CD genotype classification).

*Categorical tests for survival data (CTSD)* are used to identify significant differences between categories of individuals grouped by failure times. The right-censored survival data are required to perform categorical survival tests. The baseline null hypothesis is formulated in terms of the identity of cumulative distribution functions corresponding to different groups of individuals. Individuals involved in the analysis are classified by observed failure or censoring times according to specified rules.

It should be noted that categorical tests for survival data are not strictly applicable for testing a categorical null hypothesis formulated in terms of interval probabilities for failure times, as done in classical categorical analysis with continuous response variable. A target null hypothesis corresponding to CTSD involves censoring and lag between infection time and start of observation, as well as rules of classification. On the other hand, under mild conditions of experimental design the baseline null hypothesis implies the target null hypothesis, and therefore, rejection of the target null hypothesis implies rejection of the baseline null hypothesis.

*Hardy-Weinberg equilibrium (HWE)* tests are performed to evaluate significant deviation from Hardy–Weinberg equilibrium that is commonly used as an indicator of genotyping errors. Haldane’s exact test on Hardy–Weinberg equilibrium [3] is used to produce p-values. Sign of Hardy–Weinberg disequilibrium statistic is applied to specify direction of the disequilibrium. The **R**-function HWExact() of *HardyWeingerg* package is used to perform HWE test.

For the convenience of test results representation, several different statistics, which describe the direction and strength of association between a SNP and disease characteristic in different tests (odds ratio, relative hazard and ez2-transformed correlation coefficient), are combined under the general term of Quantitative Association Statistic (QAS). The QAS takes positive values. Values of QAS>1 and QAS<1 correspond to positive and negative associations, respectively.

**Supplementary Figures.**


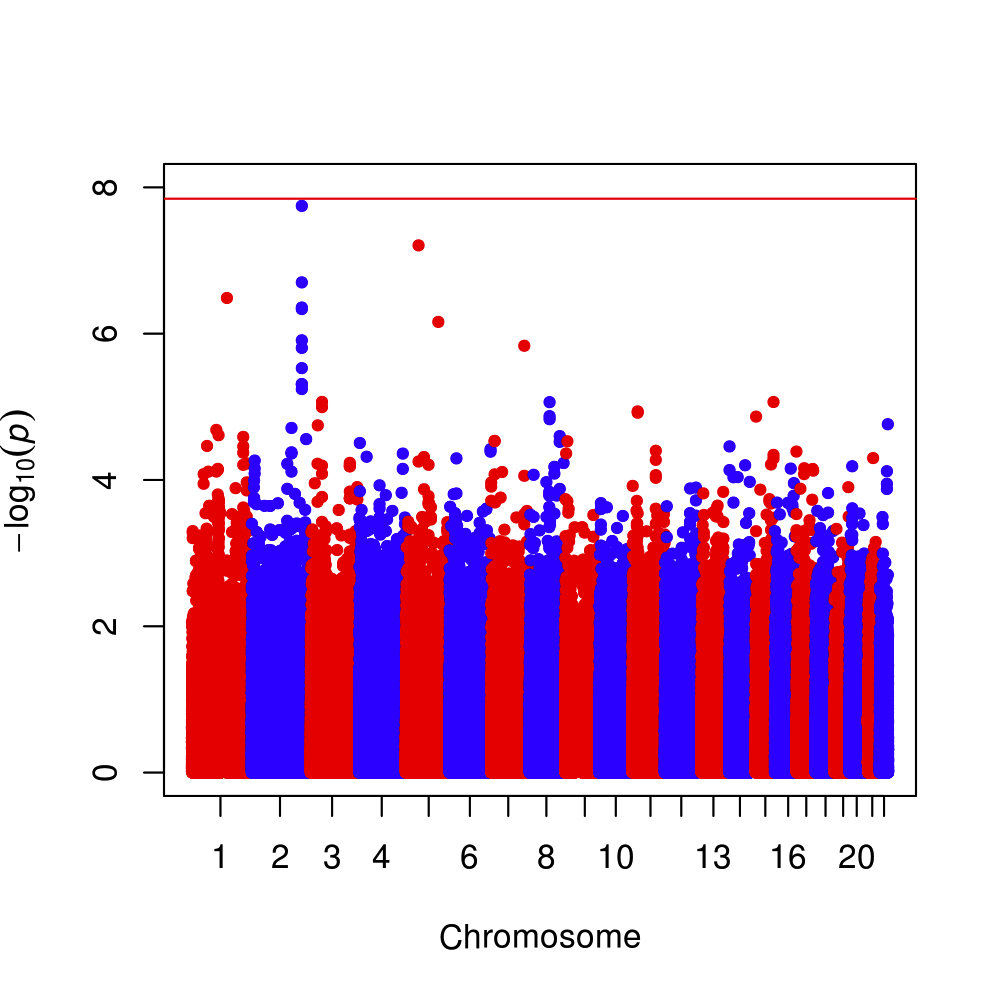


**Supplementary Figure 1. MANHATTAN** plot for one of the association tests (PHAZ/CAUCASIAN/87/D in Study Group A) that reveals SNPs in AIDS Restriction Genes.


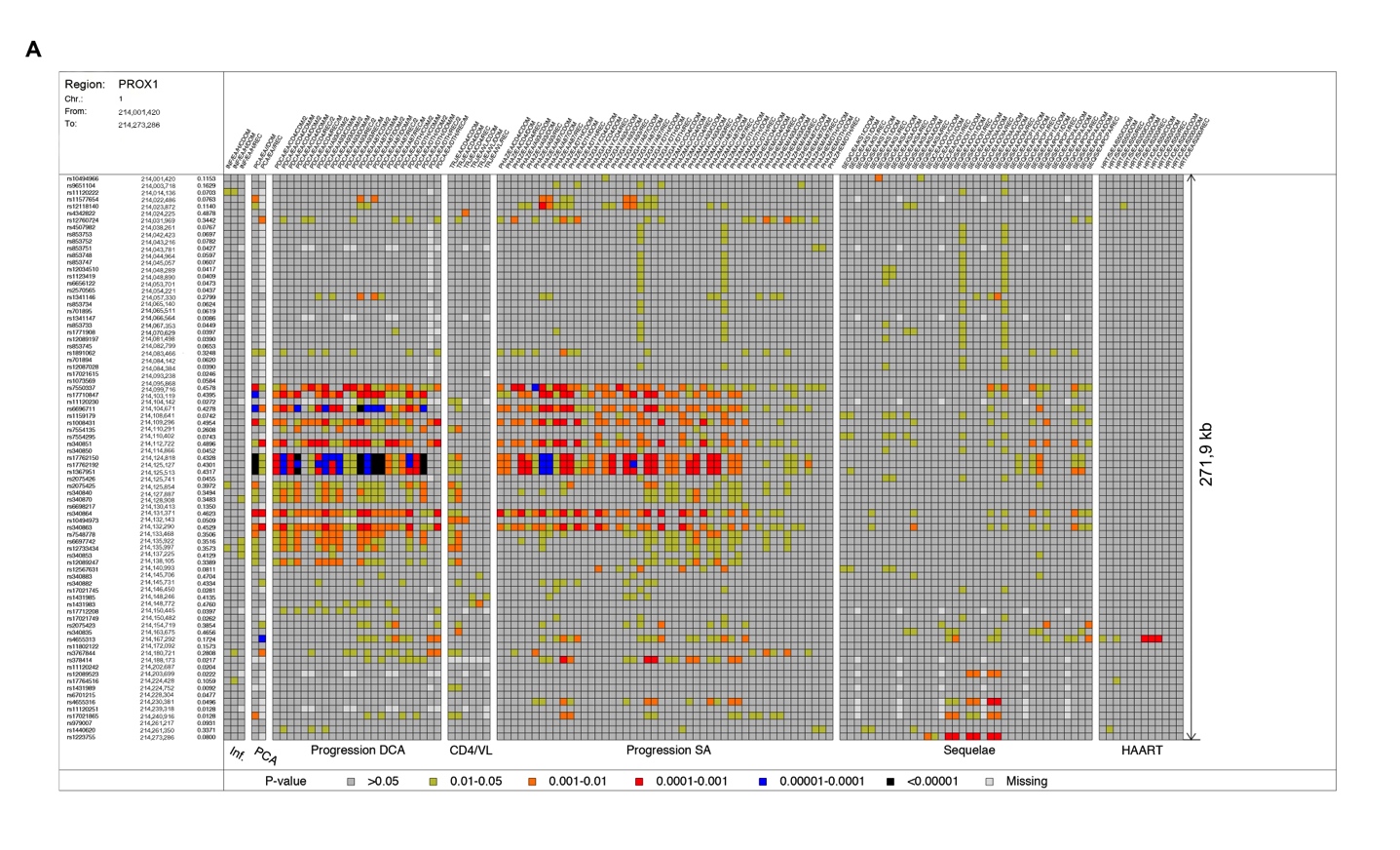

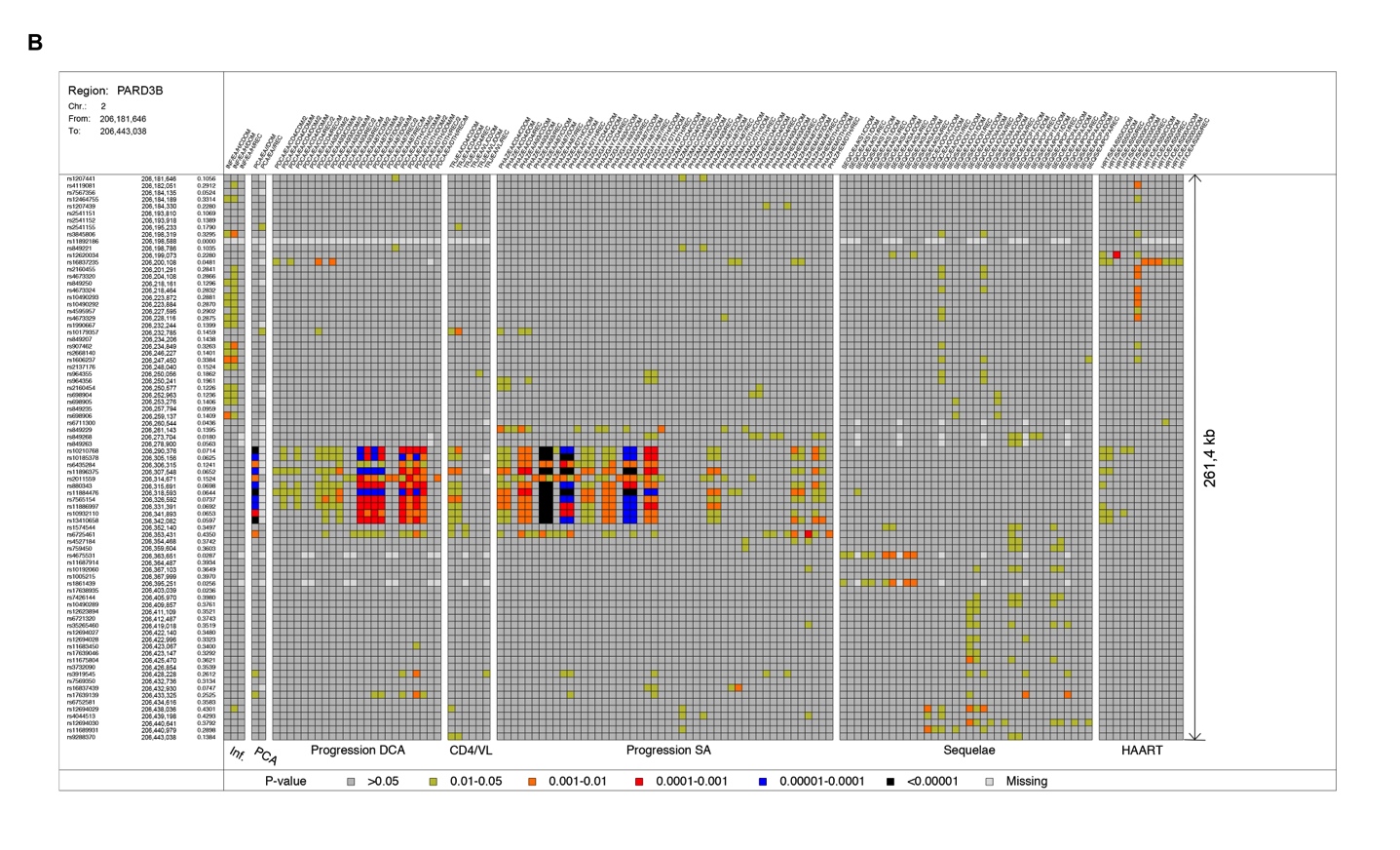

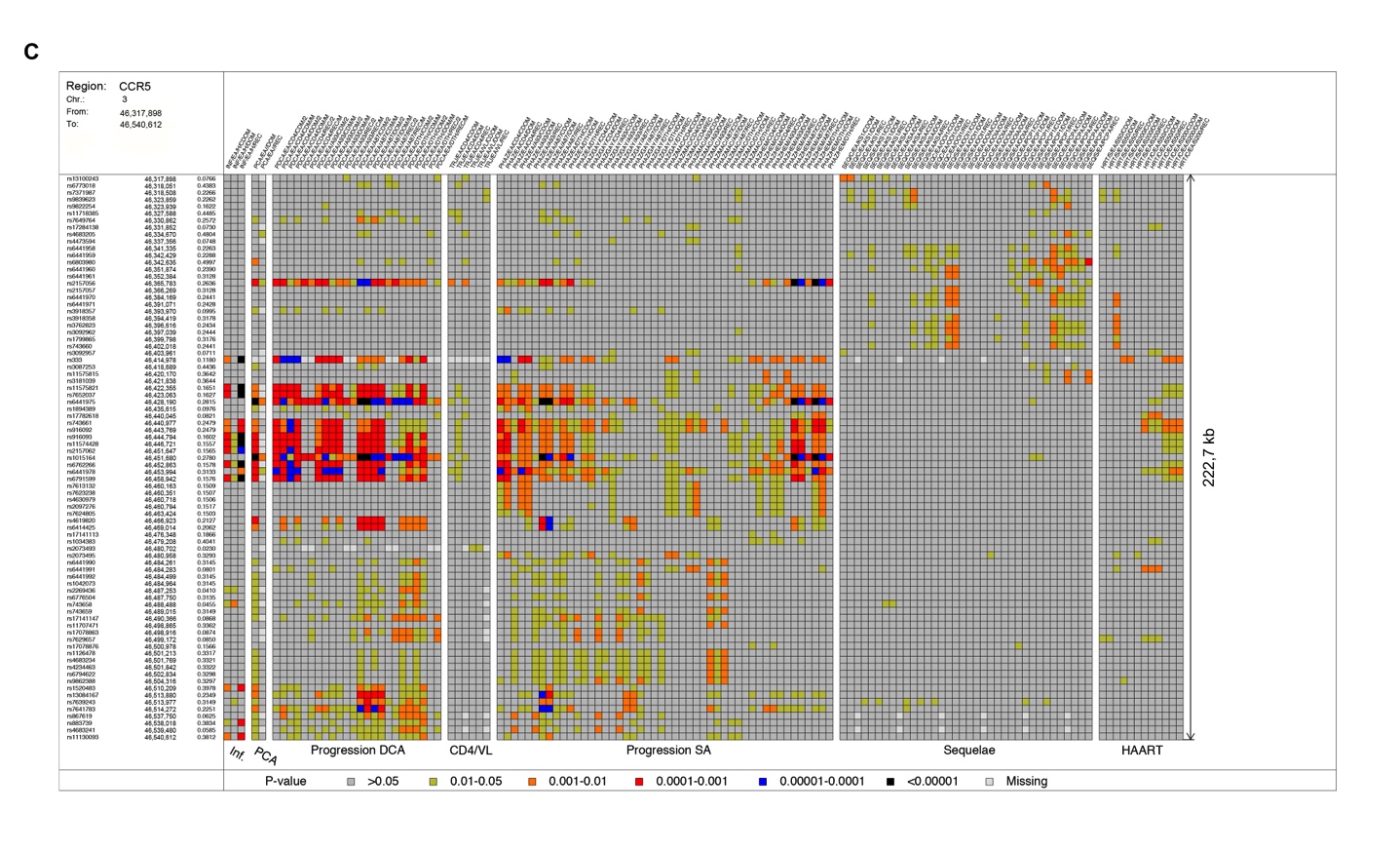


**Supplementary Figure 2**. **2D-SNAPSHOT** heat plot of selected regions illustrating significant p-values (color intensity) for association of linked SNPs alleles in: (**A**) *PROX1* (Chr. 1), (**B**) *PARD3B* (Chr. 2) and (**C**) *CCR5* (Chr. 3).


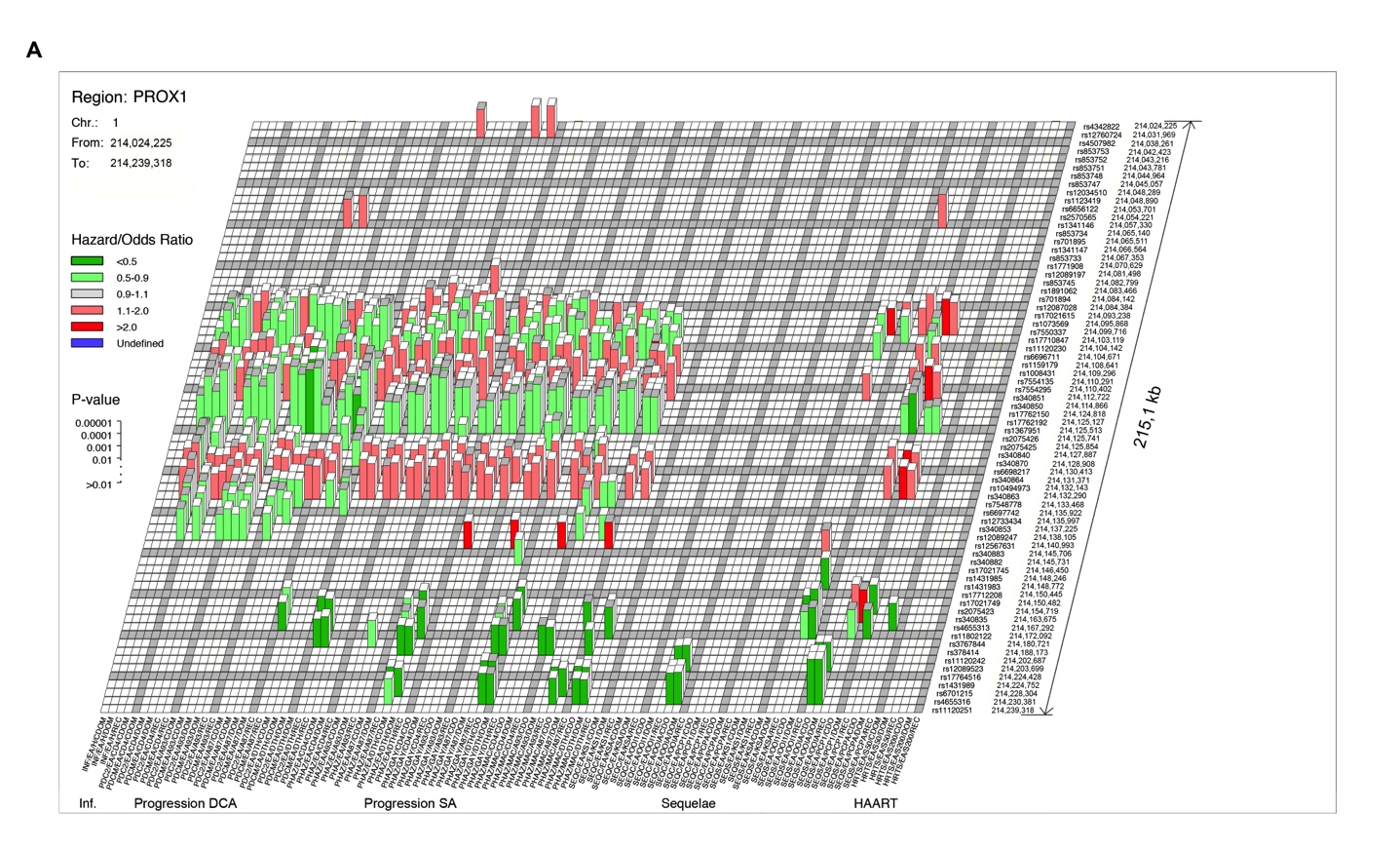

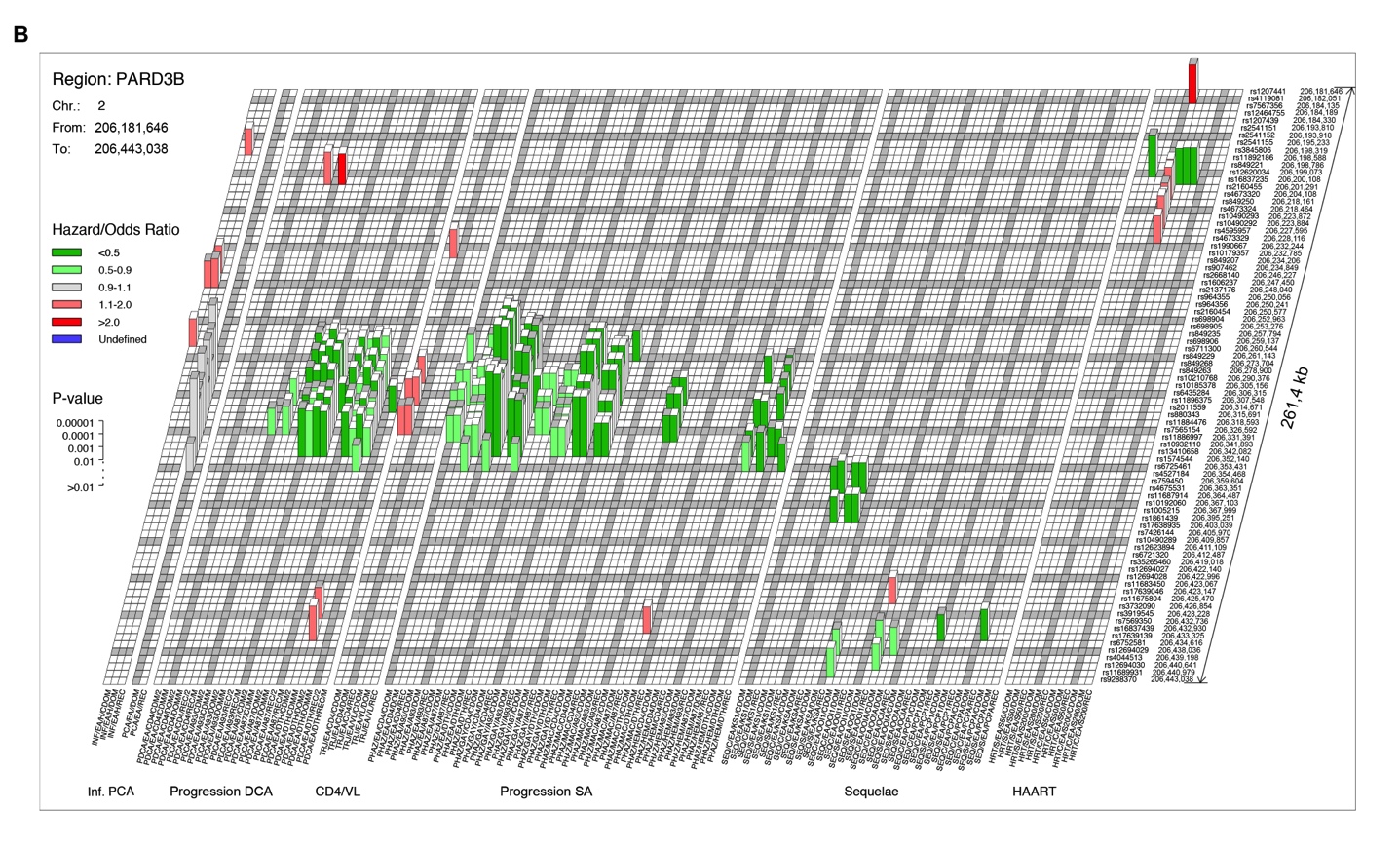

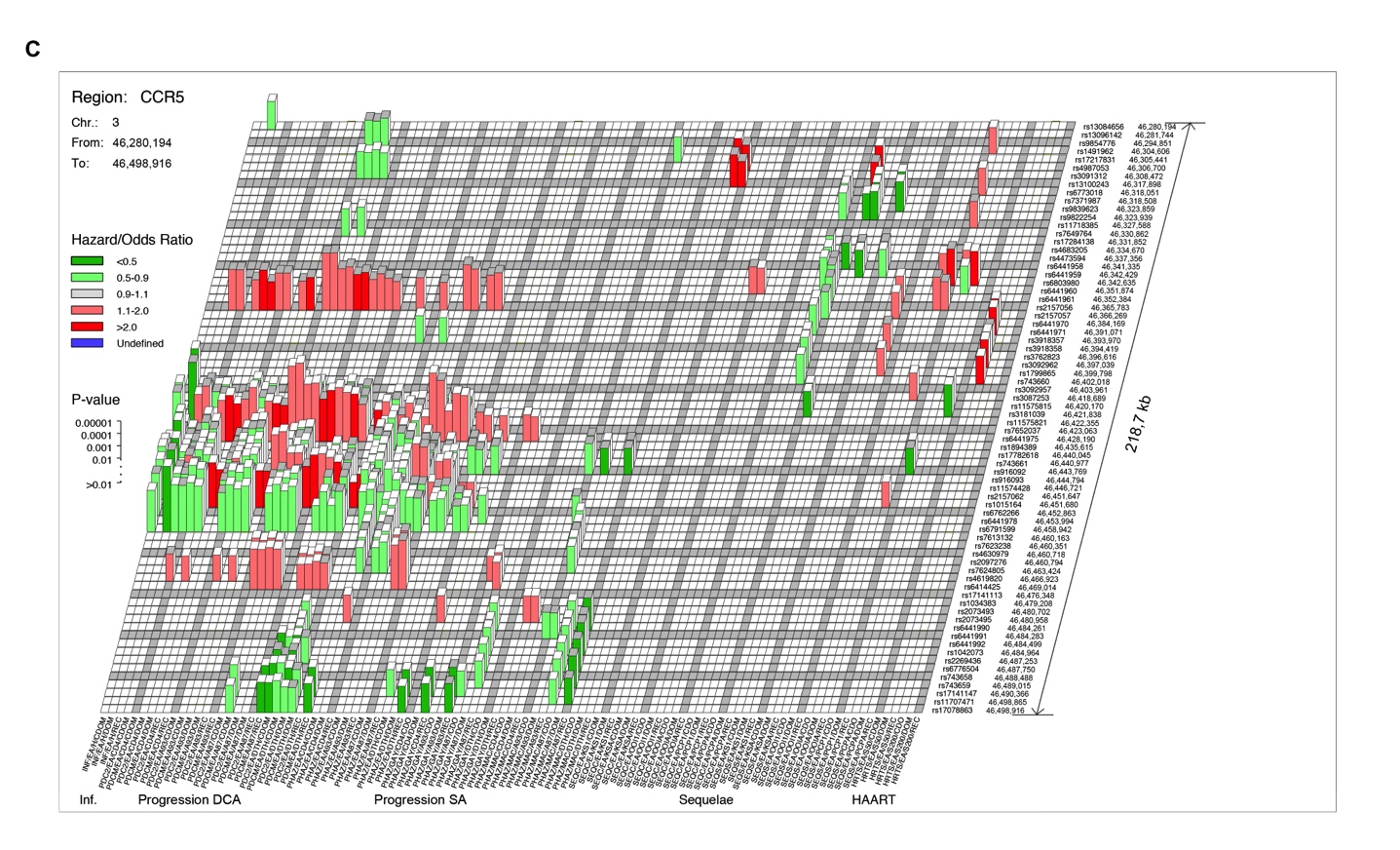


**Supplementary Figure 3. 3D-SNAPSHOT** of selected regions illustrating significant p-values (block height), QAS-based direction (color: green for QAS<1.0, red for QAS>1.0) and QAS-based strength (color intensity) of association for linked SNPs alleles in (**A**) *PROX1* (Chr. 1), (**B**) *PARD3B* (Chr. 2) and (**C**) *CCR5* (Chr. 3).


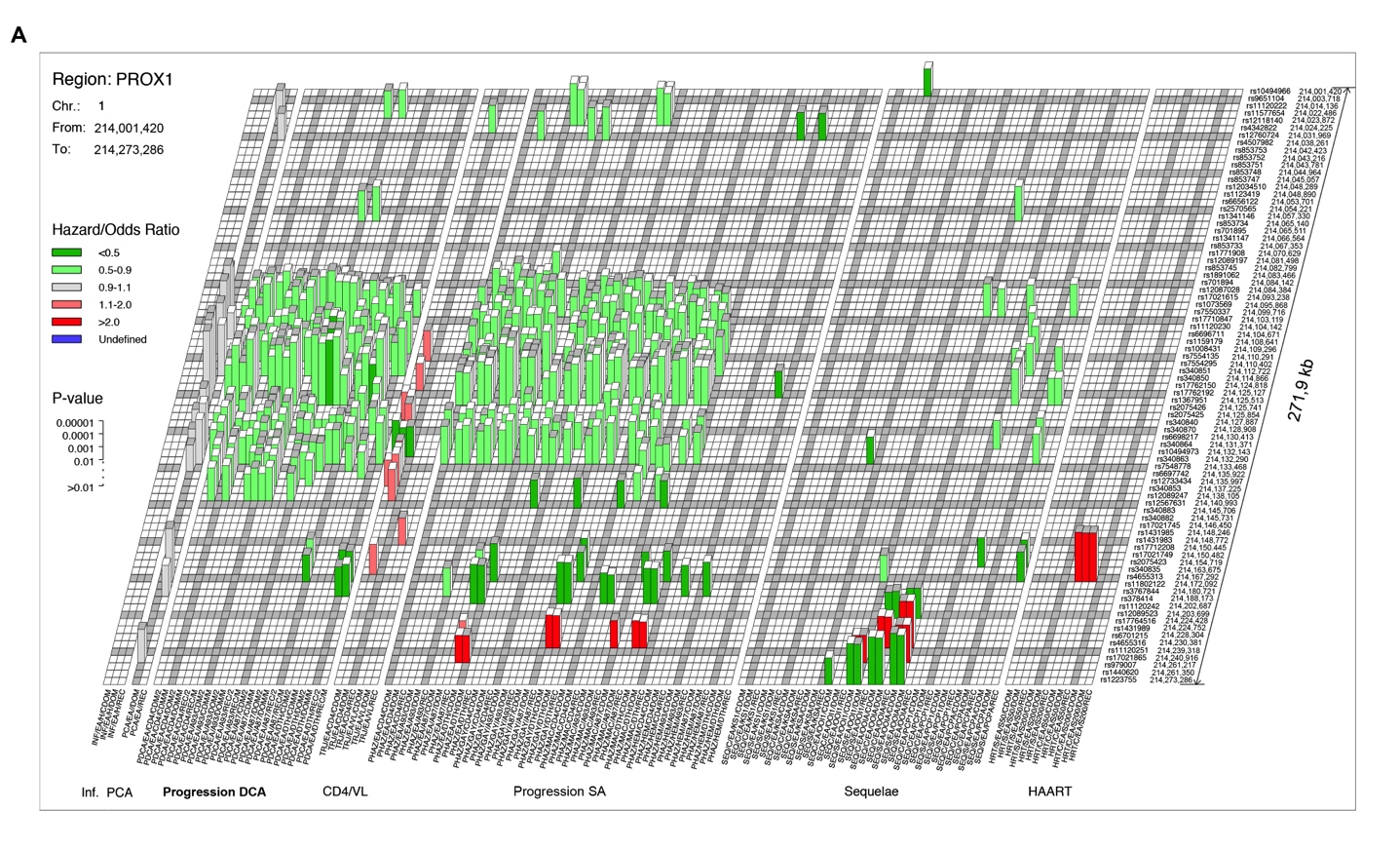

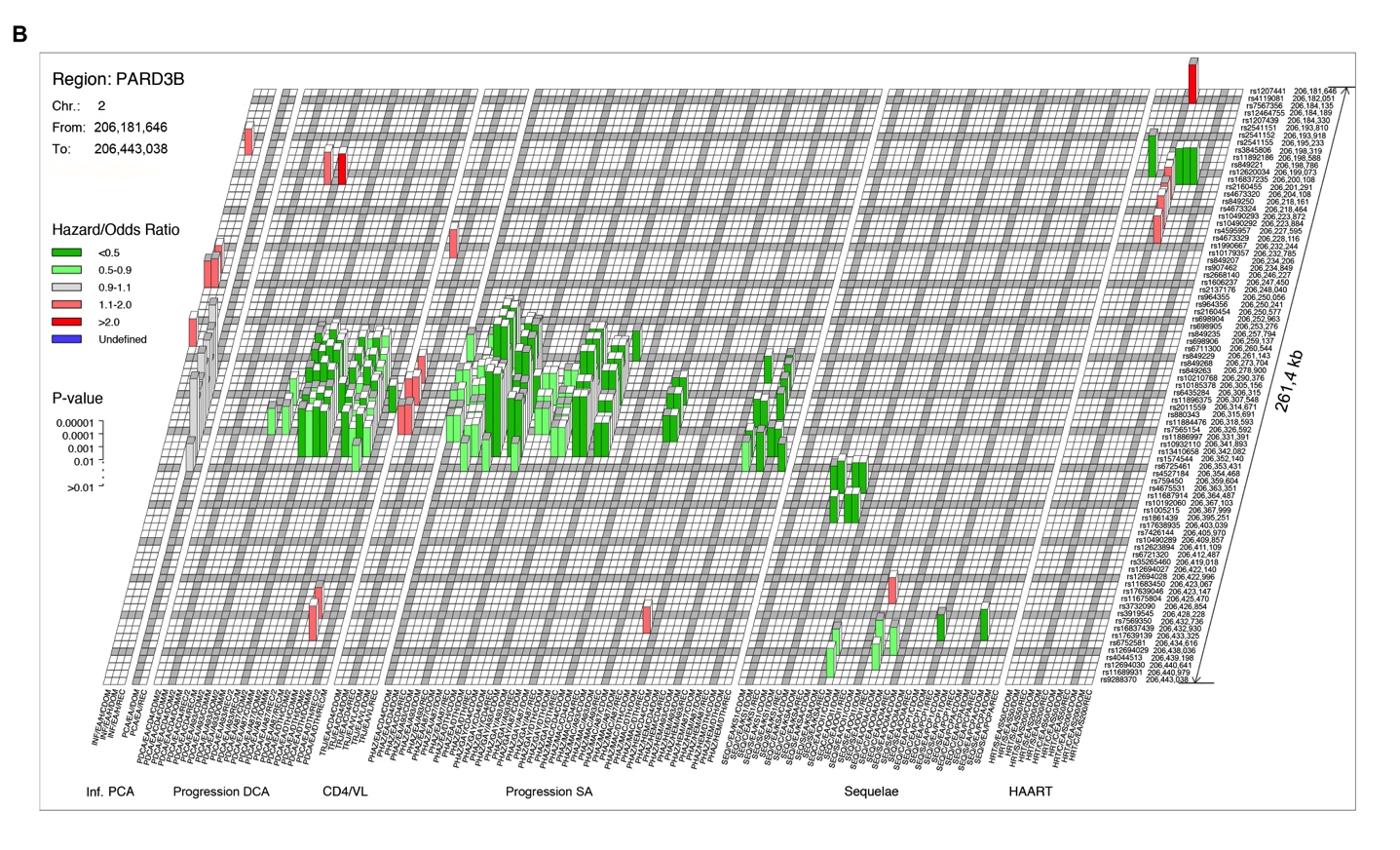

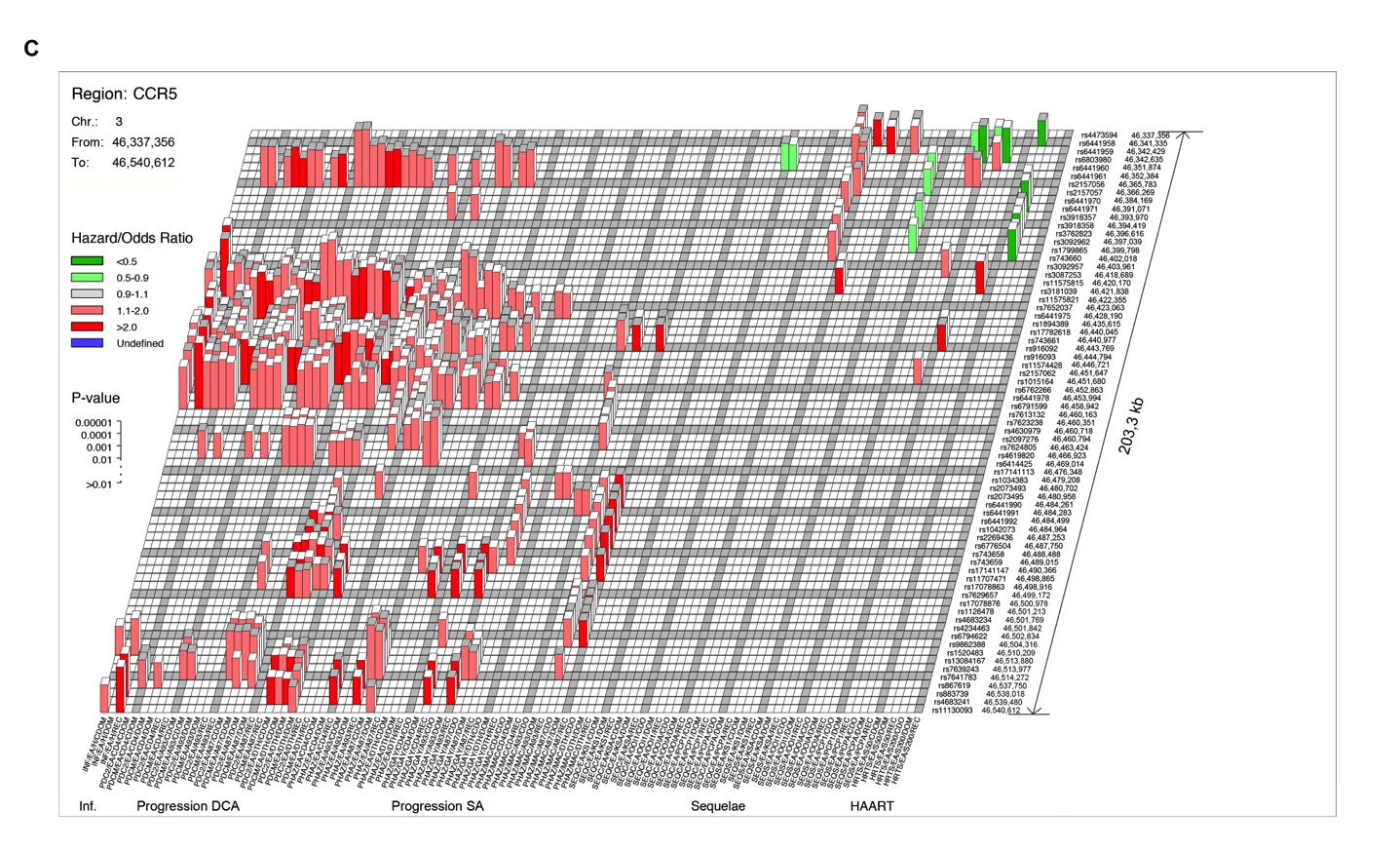


**Supplementary Figure 4. POLARIZED** **3D-SNAPSHOT** of selected regions illustrating significant p-values (block height), QAS-based direction (color: green for QAS<1.0, red for QAS>1.0) and QAS-based strength (color intensity) of association for linked SNPs alleles in (**A**) *PROX1* (Chr. 1), (**B**) *PARD3B* (Chr. 2) and (**C**) *CCR5* (Chr. 3). Polarization means the QAS values (i.e., direction of association expressed as block color) are adjusted to reflect the LD tracking of common and minor alleles at adjacent loci. This adjustment renders the colors of all proxy SNPs as the same increasing confidence that a single association signal in a gene region is driven by one causal variant tracked by proxy SNPs in LD with this causal variant.

**Supplementary Figure 5. TRAX PAGE,** a two-page summary or all test results for a single SNP for a study group (e.g., p-values and QASs for HIV infection, AIDS progression using categorical and survival tests, AIDS sequelae, and HAART outcomes can be viewed and compared). **TRAX PAGE** can be generated *de novo* for any SNP of interest by placing mouse tip over a significant tower/block in the **HIGHWAY** and selecting the TRAX PAGE option from the data window that appears (SNPs for which **TRAX REPORT** is available do not have separate TRAX PAGE option in data window since **TRAX REPORT** includes **TRAX PAGE** content).

**Supplementary Figure 6.** A detailed 11-page **TRAX REPORT** of derived statistics for all the tests accomplished including tables, bar graphs, survival curves and additional parameters for each test. **TRAX REPORT** can be generated *de novo* for the SNP of interest by placing mouse tip over a significant tower/block in **HIGHWAY** and selecting the TRAX REPORT option from the data window that appears. **TRAX REPORTs** are available for 641 SNPs in 241 human genes that were genotyped to replicate the GWAS associations for Study Groups A-C (Supplementary Table 6, in Additional File 7).

**Supplementary Tables.**

**Supplementary Table 1. Data-Table** of GWAS results: first 100 rows of the Data-Table containing SNPs, p-values and QASs for AIDS Restriction Genes in Study Group A in the *PARD3B* region of chromosome 2. Full unabridged data tables for Groups A-C are available on the web portal [gen-watch.org](file:///C:\Users\Nicole\Documents\Freelance%20work\GigaScience\MANUSCRIPTS\GWATCH\gen-watch.org).

**Supplementary Table 2.** List of SNP association statistical tests and patient counts for Study Group A.

**Supplementary Table 3.** List of SNP association statistical tests and patient counts for Study Group B.

**Supplementary Table 4.** List of SNP association statistical tests and patient counts for Study Group C.

**Supplementary Table 5.** Summary of SNP association tests performed for each Study Group.

**Supplementary Table 6.** List of 641 SNPs within 241 human genes that were assessed to replicate the GWAS associations for Study Groups A-C. For each of these SNPs a full TRAX REPORT (an 11-page report of figures and tables for each test) is available on the web portal [gen-watch.org](file:///C:\Users\Nicole\Documents\Freelance%20work\GigaScience\MANUSCRIPTS\GWATCH\gen-watch.org) as illustrated in Supplementary Figure 6 in Additional File 7.

**Supplementary Table 7.** Genomic regions of remarkable statistical association **(HITS)** identified in ARG-GWAS by the screen for extreme p-values.

**Supplementary Table 8.** QC filters for AIDS susceptibility Genes group A [4,5].

| SNP filtering | Dropped | Included |
| --- | --- | --- |
| Total SNPs |  | 934 968 |
| Supported SNPs | 28 346 | 906 622 |
| Autosomal SNPs | 35 456 | 871 166 |
| Perfect match probes | 3 009 | 868 157 |
| Passed HWE (p>0.001), editing, or visual inspection | 7 123 | 861 034 |
| Passed Mendelian inheritance | 389 | 860 645 |
| >95% genotyping call rate | 39 220 | 821 425 |
| MAF>1% | 121 403 | 700 022 |
| Total used |  | 700 022 |

**Supplementary References:**

1. Agresti A: *Categorical data analysis*, 2nd edition. Hoboken: John Wiley & Sons; 2002.
2. Cox DR: **Regression models and life tables.** *J R Stat Soc Series B Stat Methodol* 1972, **34**:187-220.
3. Haldane J: **An exact test for randomness of mating.** *J Genet* 1954, **52:**631-635.
4. Herbeck JT, Gottlieb GS, Winkler CA, Nelson GW, An P, Maust BS, Wong KG, Troyer JL, Goedert JJ, Kessing BD, Detels R, Wolinsky SM, Martinson J, Buchbinder S, Kirk GD, Jacobson LP, Margolick JB, Kaslow RA, O'Brien SJ, Mullins JI: **Multistage genomewide association study identifies a locus at 1q41 associated with rate of HIV-1 disease progression to clinical AIDS.** *J Infect Dis* 2010, **201:**618-626.
5. Troyer JL, Nelson GW, Lautenberger JA, Chinn L, McIntosh C, Johnson RC, Sezgin E, Kessing B, Malasky M, Hendrickson SL, Li G, Pontius J, Tang M, An P, Winkler CA, Limou S, Le Clerc S, Delaneau O, Zagury JF, Schuitemaker H, van Manen D, Bream JH, Gomperts ED, Buchbinder S, Goedert JJ, Kirk GD, O'Brien SJ: **Genome-wide association study implicates PARD3B-based AIDS restriction.** *J Infect Dis* 2011, **203:**1491-1502.
