# Supplementary material for: GWATCH: a web platform for automated gene association discovery analysis
Source: Gigascience. 2014 Nov 5;3:18. doi: 10.1186/2047-217X-3-18 (PMC4220276; doi:10.1186/2047-217X-3-18)
Supplement: Additional file 9: Table S6 — List of 641 SNPs within 241 human genes that were assessed to replicate the GWAS associations for Study Groups A-C. For each of these SNPs a full TRAX REPORT (11 page report of figures and tables for each test) is available on the GWATCH web portal [33] as illustrated in Additional file 8: Figure S6. [file 2047-217X-3-18-S9.pdf]

TRAX PAGE for rs11884476

Project:	ARG Group A European Americans		
Marker:	SNP_A-8393199	Maf: 0.06052	
SNP rs ID:	rs11884476		
Allele 1:	C (0.93948)	Allele 2:	G (0.06052)
Chromosome:	2	Coordinates: 206,318,593	Gene region: PARD3B

Race		Freq 1	Freq 2
Caucasian	917	0.93948	0.06052
Total	917		

	Caucasian
SC	699
OSN	81
HREU	121
SP	16
Total	917

Infection Tests

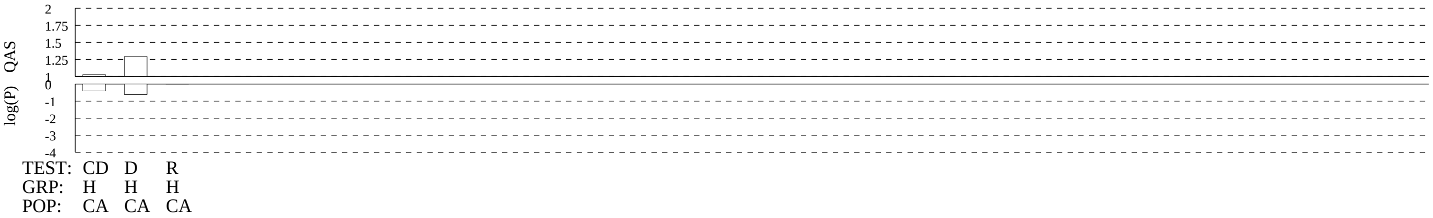

Progression disease categorical analysis (dichotomous)

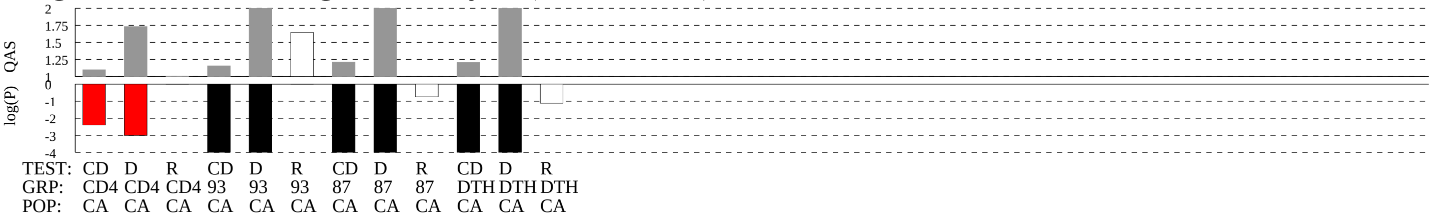

Progression disease categorical analysis (multipoint)

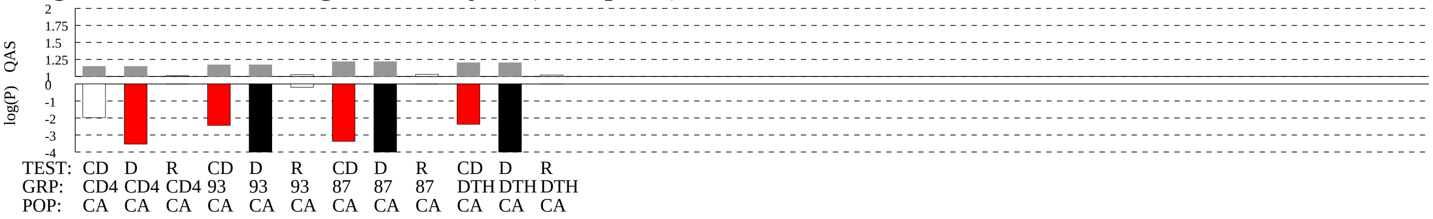

Progression survival proportional hazard analysis

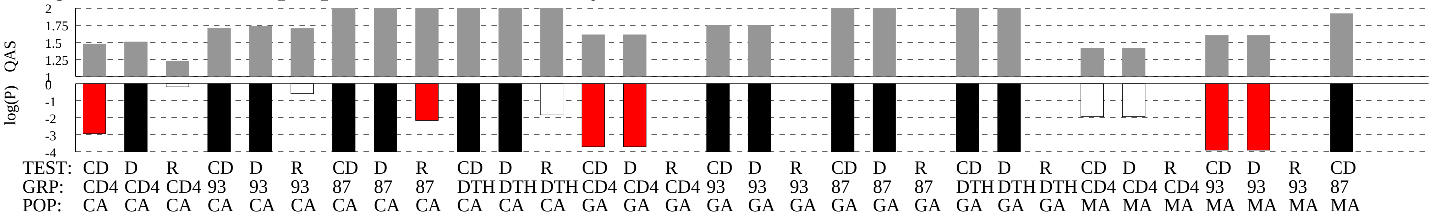

Progression survival proportional hazard analysis

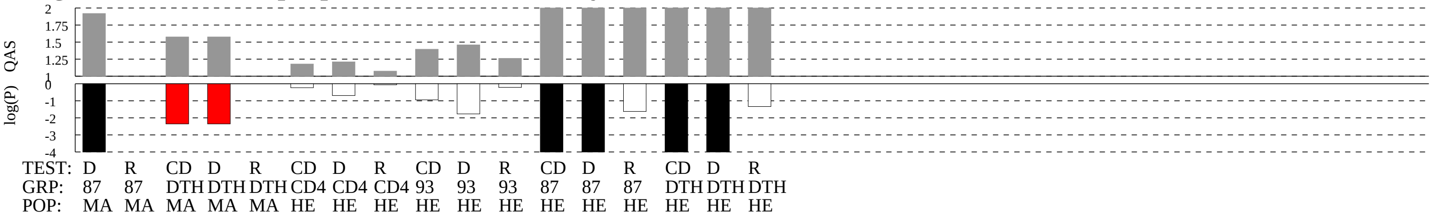

Sequelae tests (categorical) for the first infection

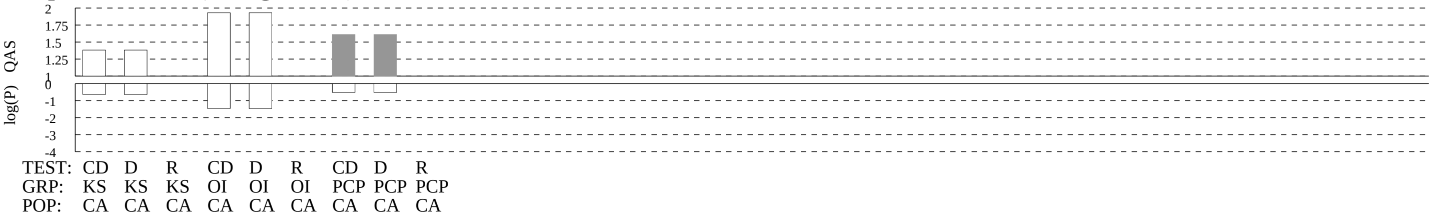

Sequelae tests (categorical) for any infection order

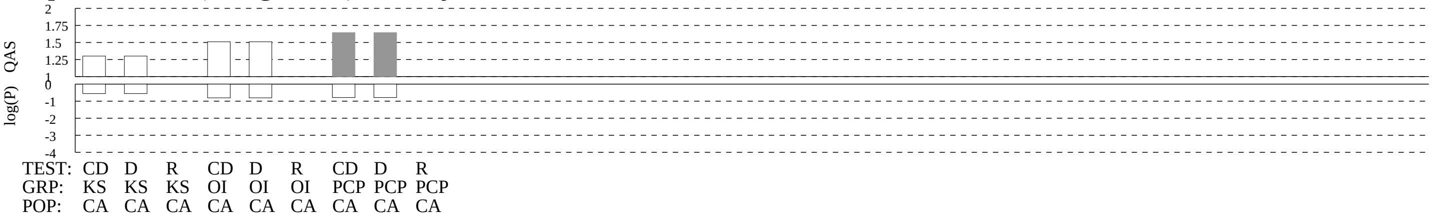

Sequelae tests (survival) for the first infection

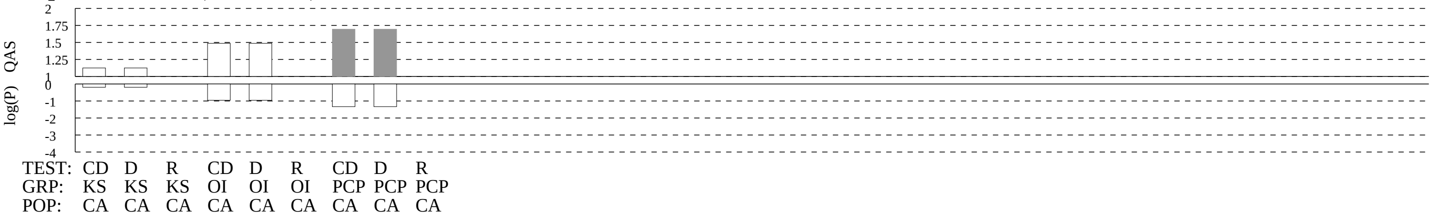

Sequelae tests (survival) for any infection order

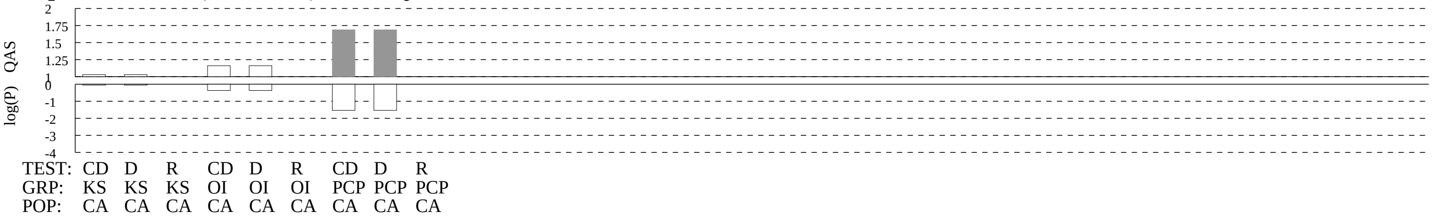

Black: QAS > 2 or -log(P) < -4

Red: -4 < -log(P) < -2

Gray: 1/QAS

White: QAS or -log(P)
