# Supplementary material for: GWATCH: a web platform for automated gene association discovery analysis
Source: Gigascience. 2014 Nov 5;3:18. doi: 10.1186/2047-217X-3-18 (PMC4220276; doi:10.1186/2047-217X-3-18)

TRAX REPORT for rs11884476

Project:	ARG Group A European Americans				
Marker:	SNP_A-8393199	Maf: 0.06052			
SNP rs ID:	rs11884476				
Allele 1:	C (0.93948)	Allele 2:	G (0.06052)		
Chromosome:	2	Coordinates:	206,318,593	Gene region:	PARD3B

Race		Freq 1	Freq 2
Caucasian	917	0.93948	0.06052
Total	917		

	Caucasian
SC	699
OSN	81
HREU	121
SP	16
Total	917

Infection Tests

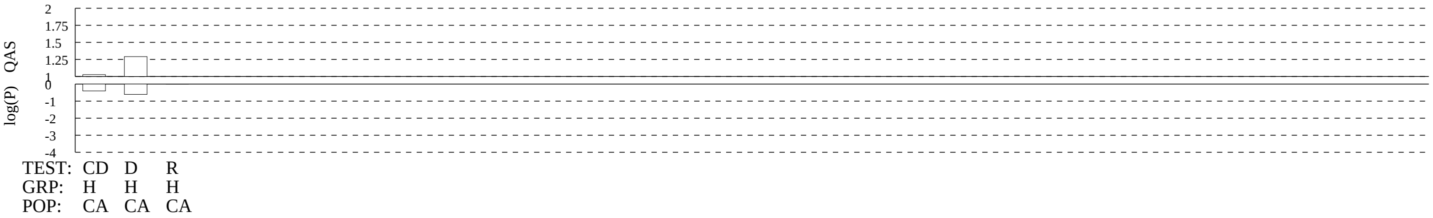

Progression disease categorical analysis (dichotomous)

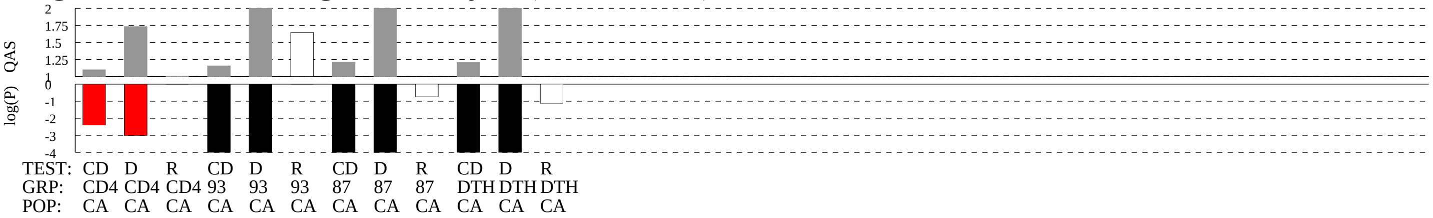

Progression disease categorical analysis (multipoint)

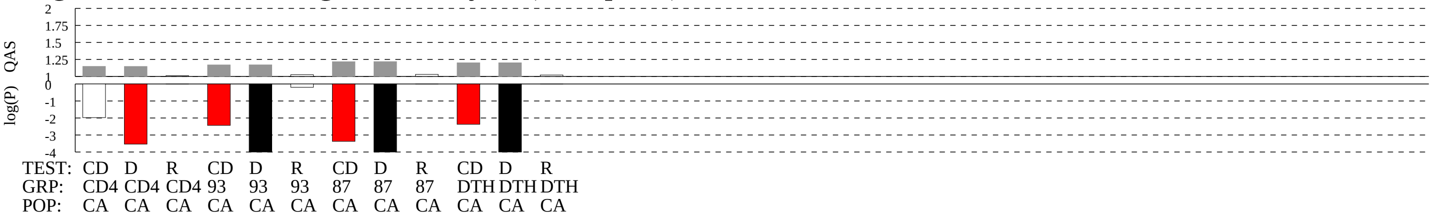

Progression survival proportional hazard analysis

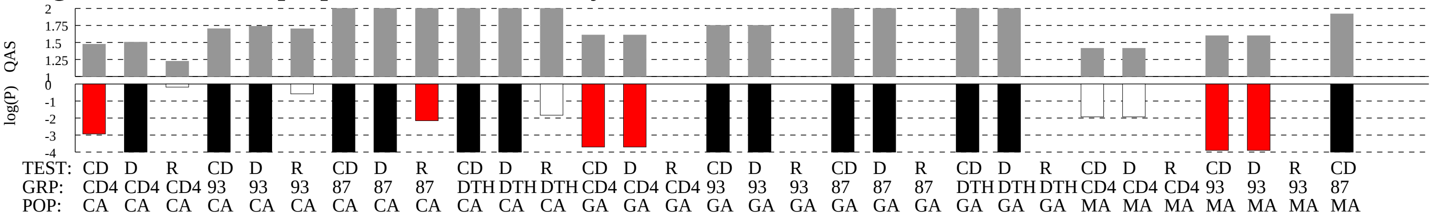

Progression survival proportional hazard analysis

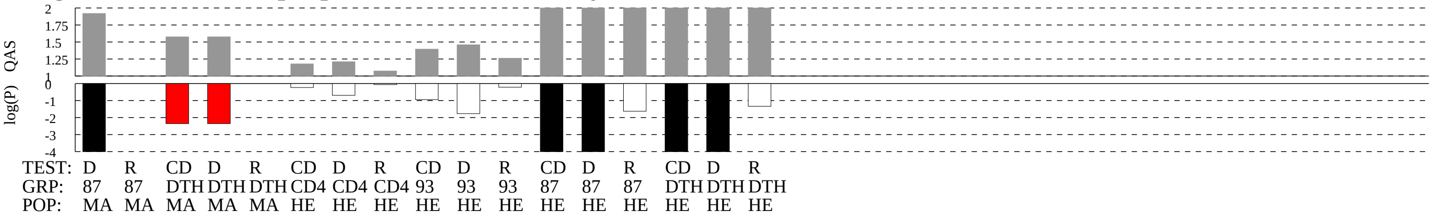

Sequelae tests (categorical) for the first infection

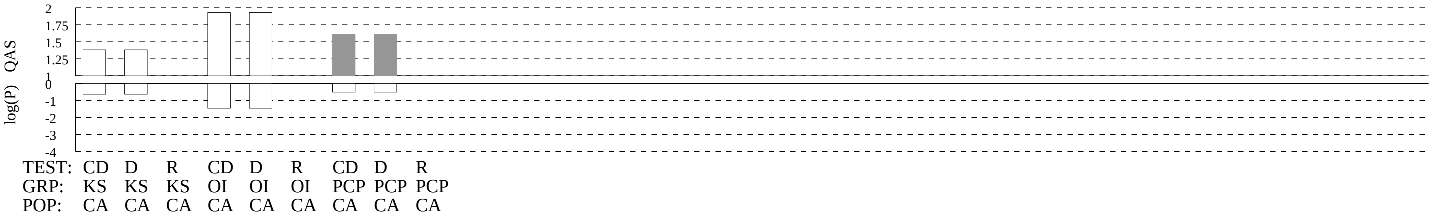

Sequelae tests (categorical) for any infection order

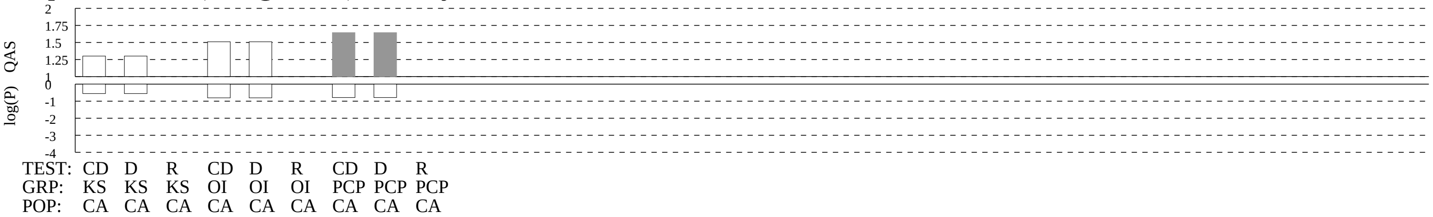

Sequelae tests (survival) for the first infection

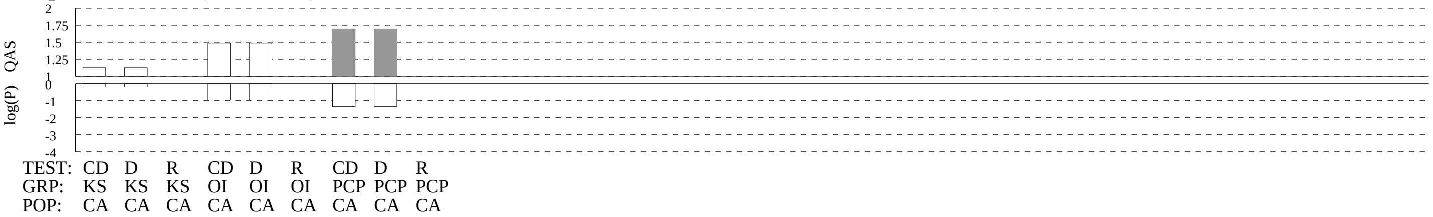

Sequelae tests (survival) for any infection order

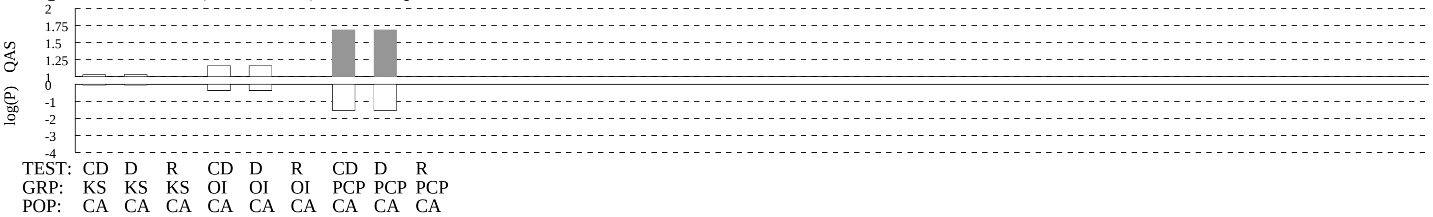

Black: QAS > 2 or -log(P) < -4

Red: -4 < -log(P) < -2

Gray: 1/QAS

White: QAS or -log(P)

Infection Tests. Genotype: black-2/2, grey-1/2

SC vs. HREU
for European American
N = 820
QAS (EZ2) = 1.026507
P = 0.668066597

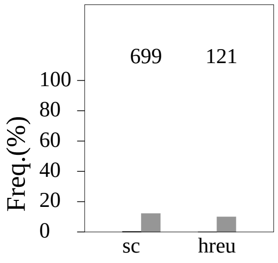

Progression disease categorical analysis (dichotomous). Genotype: black-2/2, grey-1/2

AIDS-87
for European American
N = 443
QAS (EZ2) = 0.826199
P = 4.9998E-5

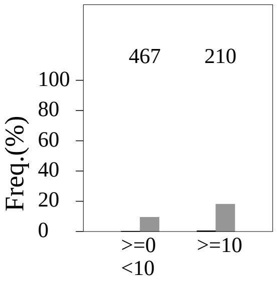

AIDS-93
for European American
N = 513
QAS (EZ2) = 0.864399
P = 0.001899905

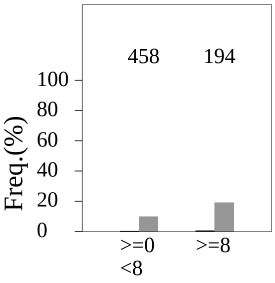

CD4 < 200
for European American
N = 489
QAS (EZ2) = 0.909601
P = 0.090745463

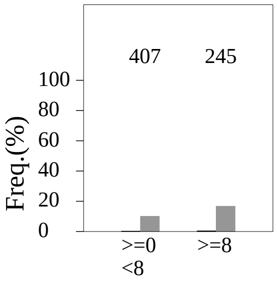

Death from AIDS
for European American
N = 328
QAS (EZ2) = 0.829081
P = 0.00119994

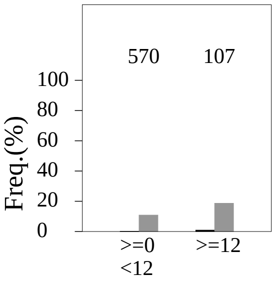

Progression disease categorical analysis (multipoint). Genotype: black-2/2, grey-1/2

AIDS-87
for European American
N = 443
QAS (EZ2) = 0.821867
P = 0.034248288

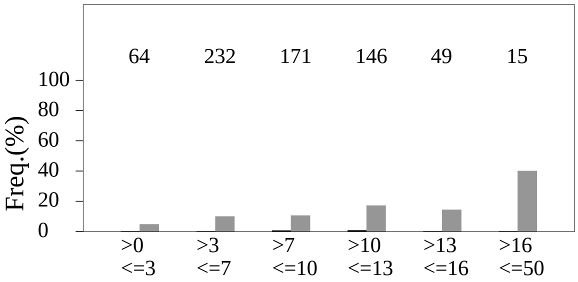

AIDS-93
for European American
N = 474
QAS (EZ2) = 0.855408
P = 0.087545623

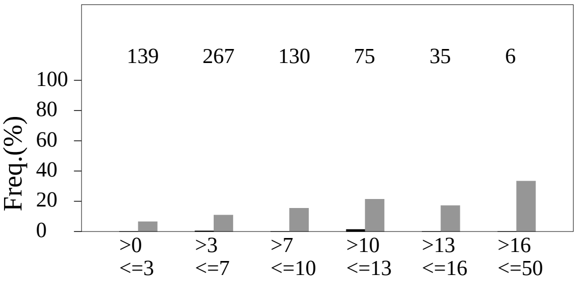

CD4 < 200
for European American
N = 432
QAS (EZ2) = 0.8718
P = 0.138443078

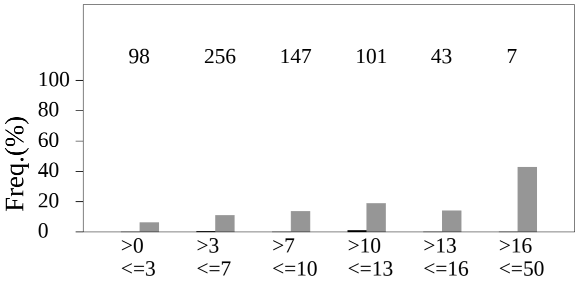

Death from AIDS
for European American
N = 435
QAS (EZ2) = 0.833354
P = 0.093295335

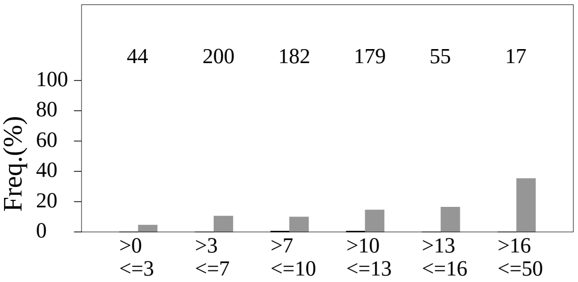

Progression survival proportional hazard analysis. Genotype: blue-1/1, red-1/2, black-2/2

CD4 < 200
for European American
N = 636
QAS (RH) = 0.678448
P = 0.053206094

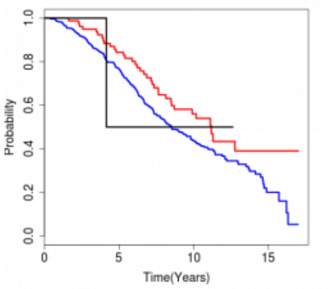

CD4 < 200
for Gays
N = 470
QAS (RH) = 0.621373
P = 0.02469109

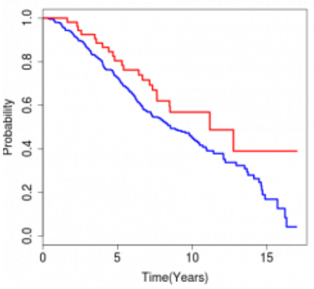

AIDS-93
for European American
N = 636
QAS (RH) = 0.587542
P = 0.001405878

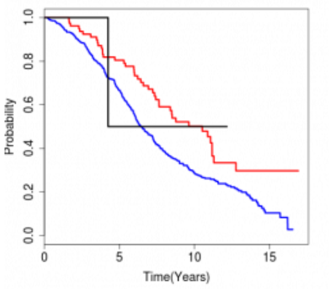

AIDS-93
for Gays
N = 470
QAS (RH) = 0.571219
P = 0.002438975

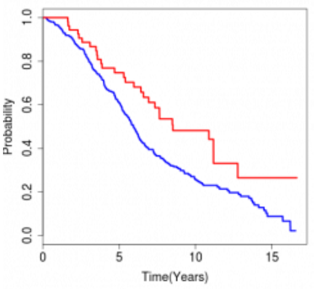

AIDS-87
for European American
N = 661
QAS (RH) = 0.363486
P = 4.703E-6

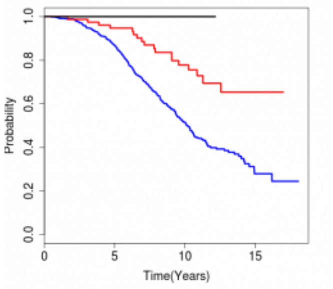

AIDS-87
for Gays
N = 476
QAS (RH) = 0.399621
P = 0.000253772

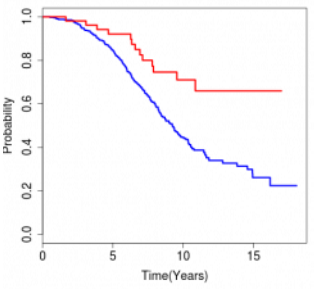

Death from AIDS
for European American
N = 661
QAS (RH) = 0.408697
P = 0.000162005

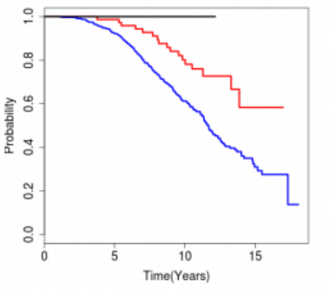

Death from AIDS
for Gays
N = 476
QAS (RH) = 0.4897
P = 0.006043265

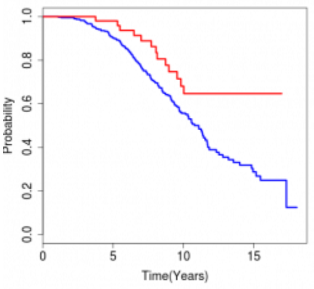

CD4 < 200
for Cohort MACS
N = 397
QAS (RH) = 0.707536
P = 0.145166367

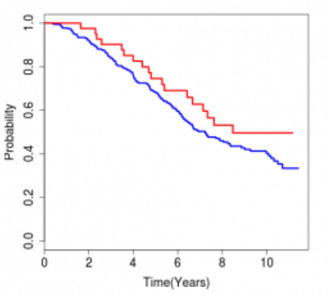

CD4 < 200
for Hemophiliac
N = 155
QAS (RH) = 0.848749
P = 0.79538435

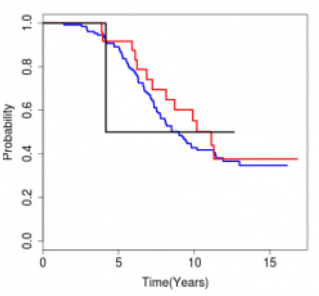

AIDS-93
for Cohort MACS
N = 397
QAS (RH) = 0.625143
P = 0.020208981

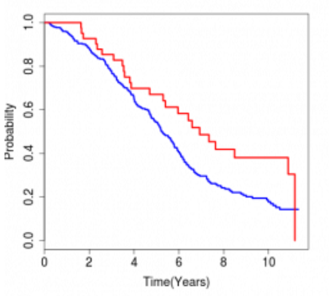

AIDS-93
for Hemophiliac
N = 155
QAS (RH) = 0.716545
P = 0.390470473

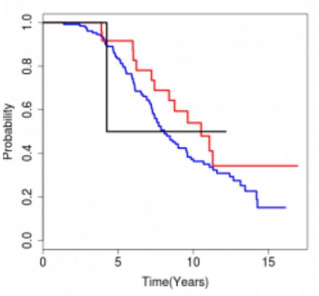

AIDS-87
for Cohort MACS
N = 401
QAS (RH) = 0.521068
P = 0.013155699

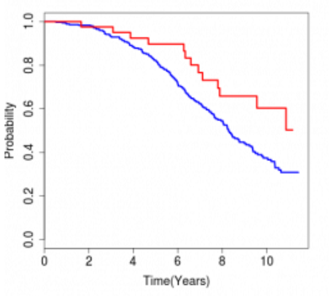

AIDS-87
for Hemophiliac
N = 173
QAS (RH) = 0.332207
P = 0.012499368

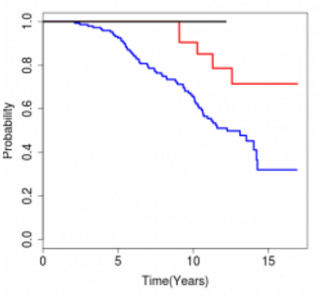

Death from AIDS
for Cohort MACS
N = 401
QAS (RH) = 0.634683
P = 0.09521055

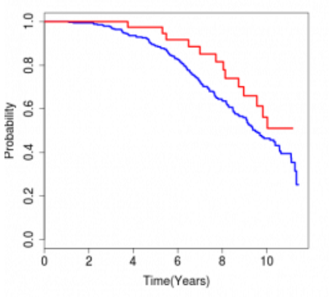

Death from AIDS
for Hemophiliac
N = 173
QAS (RH) = 0.298948
P = 0.013720821

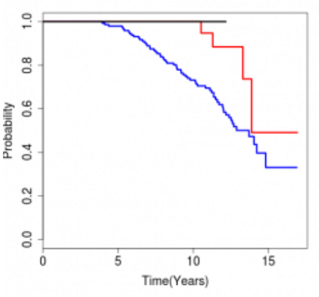

Sequelae tests (categorical) for the first infection . Genotype: black-2/2, grey-1/2

Kaposis sarcoma
for European American
N = 281
QAS (EZ2) = 1.382376
P = 0.529649413

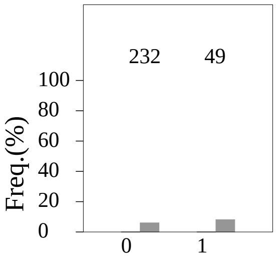

Other opport. inf.
for European American
N = 281
QAS (EZ2) = 1.929997
P = 0.23259811

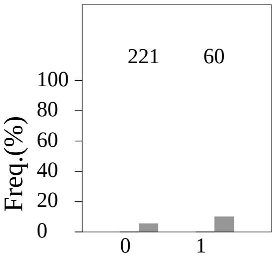

Pneumocystis Pneumonia
for European American
N = 281
QAS (EZ2) = 0.620587
P = 0.59889701

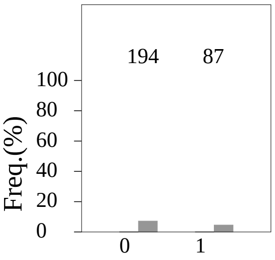

Sequelae tests (categorical) for any infection order . Genotype: black-2/2, grey-1/2

Kaposis sarcoma
for European American
N = 281
QAS (EZ2) = 1.299988
P = 0.575004399

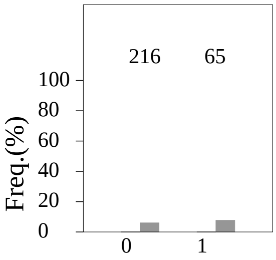

Other opport. inf.
for European American
N = 281
QAS (EZ2) = 1.509821
P = 0.447667787

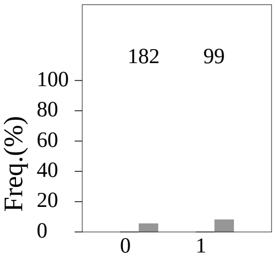

Pneumocystis Pneumonia
for European American
N = 281
QAS (EZ2) = 0.608102
P = 0.455269145

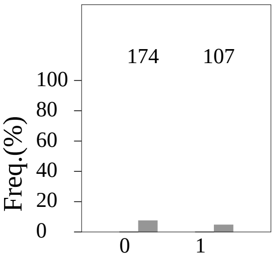

Sequelae tests (survival) for the first infection . Genotype: blue-1/1, red-1/2, black-2/2

Kaposis sarcoma
for European American
N = 278
QAS (RH) = 1.124221
P = 0.825825465

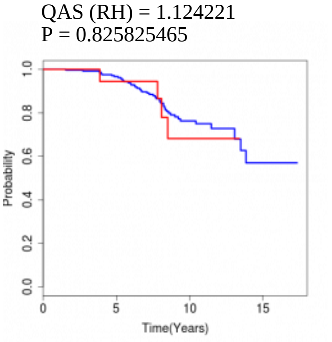

Other opport. inf.
for European American
N = 278
QAS (RH) = 1.485982
P = 0.384441579

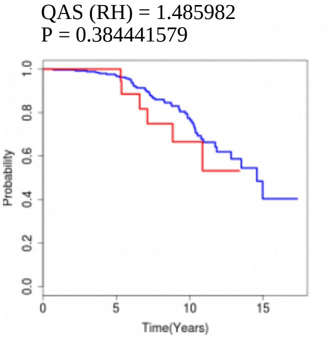

Pneumocystis Pneumonia
for European American
N = 278
QAS (RH) = 0.590162
P = 0.264004295

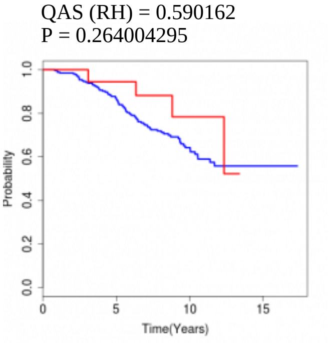

Sequelae tests (survival) for any infection order. Genotype: blue-1/1, red-1/2, black-2/2

Kaposis sarcoma
for European American
N = 278
QAS (RH) = 1.030106
P = 0.949568421

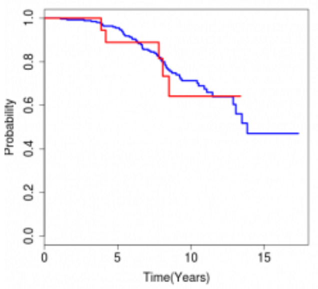

Other opport. inf.
for European American
N = 278
QAS (RH) = 1.160841
P = 0.693214379

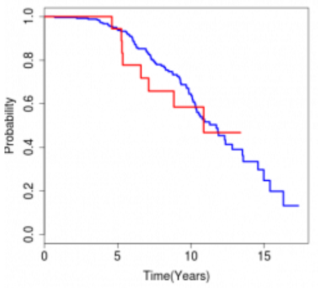

Pneumocystis Pneumonia
for European American
N = 278
QAS (RH) = 0.59254
P = 0.215783117

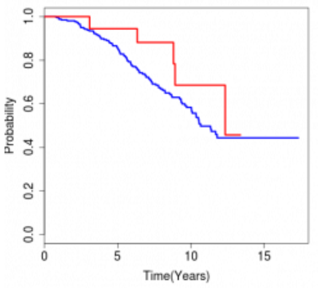

Supplement: Additional file 10: Table S7 — Genomic regions of remarkable statistical association (HITS) identified in ARG-GWAS by the screen for extreme p-values. [file 2047-217X-3-18-S10.pdf]
